# Interplay of Neuroinflammation and Gut Microbiota Dysbiosis in Alzheimer’s Disease Using Diffusion Kurtosis Imaging Biomarker in 3 × Tg-AD Mouse Models

**DOI:** 10.1021/acschemneuro.5c00063

**Published:** 2025-04-08

**Authors:** Lalitha Palanivelu, Ching-Wen Chang, Ssu-Ju Li, Yao-Wen Liang, Yu-Chun Lo, You-Yin Chen

**Affiliations:** † International Ph.D. Program in Medicine, College of Medicine, Taipei Medical University, 7F., No. 250, Wuxing Street, Xinyi District, Taipei 11031, Taiwan; ‡ Department of Biomedical Engineering, National Yang Ming Chiao Tung University, No. 155, Sec. 2, Linong Street, Taipei 112304, Taiwan; § Ph.D. Program in Medical Neuroscience, College of Medical Science and Technology, Taipei Medical University, 12F., Education and Research Building, Shuang-Ho Campus, No. 301, Yuantong Road, New Taipei City 23564, Taiwan

**Keywords:** gut-brain axis, Alzheimer’s disease, neuroinflammation, diffusion kurtosis imaging, gut microbiota, short-chain fatty acids

## Abstract

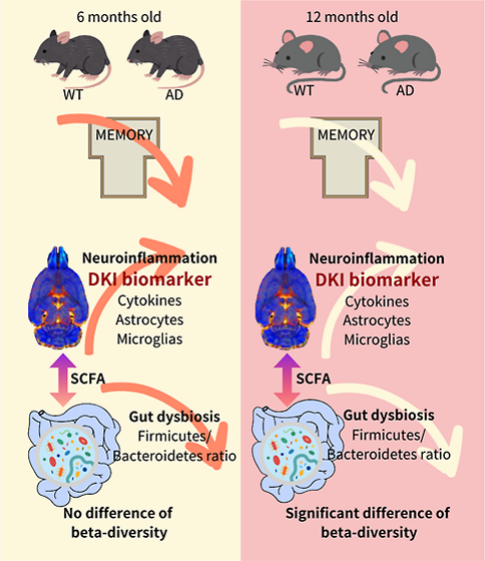

The relationship between alterations in brain microstructure and dysbiosis of gut microbiota in Alzheimer’s disease (AD) has garnered increasing attention, although the functional implications of these changes are not yet fully elucidated. This research examines how neuroinflammation, systemic inflammation, and gut microbiota interact in male 3 × Tg-AD and B6129SF1/J wild-type (WT) mice at 6 months-old (6-MO) and 12 months-old (12-MO). Employing a combination of behavioral assessments, diffusion kurtosis imaging (DKI), microbiota profiling, cytokine analysis, short-chain fatty acids (SCFAs), and immunohistochemistry, we explored the progression of AD-related pathology. Significant memory impairments in AD mice at both assessed ages were correlated with altered DKI parameters that suggest neuroinflammation and microstructural damage. We observed elevated levels of pro-inflammatory cytokines, such as IL-1β, IL-6, TNFα, and IFN-γ, in the serum, which were associated with increased activity of microglia and astrocytes in brain regions critical for memory. Although gut microbiota analysis did not reveal significant changes in alpha diversity, it did show notable differences in beta diversity and a diminished *Firmicutes*/*Bacteroidetes* (*F*/*B*) ratio in AD mice at 12-MO. Furthermore, a reduction in six kinds of SCFAs were identified at two time points of 6-MO and 12-MO, indicating widespread disruption in gut microbial metabolism. These findings underscore a complex bidirectional relationship between systemic inflammation and gut dysbiosis in AD, highlighting the gut-brain axis as a crucial factor in disease progression. This study emphasizes the potential of integrating DKI metrics, microbiota profiling, and SCFA analysis to enhance our understanding of AD pathology and to identify new therapeutic targets.

## Introduction

Alzheimer’s disease (AD) is a progressive neurodegenerative disorder characterized by cognitive decline, memory impairment, and neurological dysfunction. While traditional research has primarily focused on central nervous system (CNS) features, such as amyloid-beta (Aβ) plaques and tau protein hyperphosphorylation, emerging studies reveal the significance of peripheral factors, including systemic inflammation, gut dysbiosis, and gut-brain axis disruption, in disease progression.^1,2^ Exploring the interactions between these peripheral contributors and CNS pathology offers a more integrated understanding of AD and the potential for innovative diagnostic and therapeutic approaches.

The accumulation of Aβ is one of the earliest pathological events in AD and is closely linked to astrocyte activation through the nuclear factor kappa B (NF-κB) pathway. This activation induces the release of complement C3, a critical mediator of neuroinflammation.^3^ C3 binds to C3a receptors expressed on microglia and neurons, driving microglial activation and neuronal dysfunction.^3,4^ Simultaneously, microglia secrete pro-inflammatory mediators, including interleukin-1 alpha (IL-1α), tumor necrosis factor (TNF), and complement component 1q (C1q). These mediators contribute to the formation of A1 neurotoxic astrocytes, which are implicated in synaptic impairment and neuronal degeneration.^3,5^

This establishes a positive feedback loop involving neuroinflammation, microgliosis, and astrogliosis, leading to key pathological changes, including blood–brain barrier (BBB) dysfunction, impaired microcirculation, and neuronal loss.^6,7^ Such alterations exacerbate AD progression, facilitating the transition from mild cognitive impairment (MCI) to more severe stages of dementia.^8,9^ Chronic neuroinflammation compromises the BBB’s integrity, allowing peripheral inflammatory molecules to infiltrate the CNS and amplify neuroinflammatory responses. Additionally, BBB dysfunction enables the leakage of CNS-derived inflammatory signals into systemic circulation, establishing a bidirectional relationship between central and peripheral inflammation. This interaction amplifies the neuroinflammatory cascade, contributing to disease progression.

Systemic inflammation also plays a pivotal role in AD pathology. Elevated levels of pro-inflammatory cytokines, including interleukin-1 beta (IL-1β), interleukin-6 (IL-6), tumor necrosis factor alpha (TNFα), and interferon-gamma (IFN-γ), exacerbate neuroinflammation and neuronal dysfunction.^10^ These cytokines interact with peripheral systems, such as the gut microbiota, further highlighting the interconnectedness of systemic and CNS pathology in AD.

Gut dysbiosis, defined as an imbalance in gut microbial composition and function, has emerged as a significant contributor to AD pathogenesis. Both clinical studies and animal models of AD report alterations in gut microbial diversity, particularly a reduction in the *Firmicutes*-to-*Bacteroidetes* (*F*/*B*) ratio.^11−13^ Such imbalances compromise gut barrier function, resulting in increased intestinal permeability, or “leaky gut,” which allows microbial components such as lipopolysaccharides (LPS) to enter systemic circulation.^14^ LPS and other microbial products stimulate systemic inflammation, which exacerbates neuroinflammation and accelerates AD progression.

Short-chain fatty acids (SCFAs), metabolites produced by gut bacteria, play a critical role in maintaining intestinal integrity, modulating systemic immunity, and supporting CNS homeostasis.^15^ Dysbiosis reduces SCFA production, fostering a pro-inflammatory environment and increasing gut permeability.^16−18^ SCFAs such as acetate, propionate, and butyrate are essential for enhancing gut barrier function, suppressing systemic inflammation, and regulating neuroinflammatory pathways. In AD models, reduced SCFA levels have been associated with increased gut permeability, heightened systemic inflammation, and diminished neuroprotection.^16−18^ Moreover, specific microbial taxa within the *Firmicutes* and *Bacteroidetes* phyla are implicated in these changes. *Firmicutes* are known for their SCFA-producing capabilities, whereas certain *Bacteroidetes* species contribute to inflammatory states by producing metabolites that activate immune pathways.^19^

Neuroinflammation and its effects on brain microstructure are central to AD pathology. Advanced imaging techniques such as DKI provide valuable insights into these microstructural changes.^20^ DKI metrics, including mean kurtosis (MK), radial kurtosis (RK), and axial kurtosis (AK), are particularly sensitive to alterations associated with inflammation, gliosis, and axonal degeneration.^21^ These metrics have shown potential in detecting early microstructural changes in brain regions, such as the hippocampus (HIPP), which are critical for memory and cognitive functions.^22^

Recent findings underscore the utility of DKI in tracking AD progression. Significant reductions in kurtosis values have been observed in white matter regions of interest (ROIs) in AD patients,^23^ correlating with cognitive performance on assessments such as the mini-mental state examination (MMSE).^24^ Preclinical studies demonstrate that DKI is sensitive to microstructural changes even in the early stages of disease, making it a promising tool for noninvasive monitoring of neuroinflammation and its impact on brain integrity.^25^

While most prior research has focused on isolated aspects of AD pathology, such as CNS or gut alterations, this study emphasizes their interconnectedness. It examines the bidirectional relationship between neuroinflammation, BBB dysfunction, systemic inflammation, and gut dysbiosis. By integrating DKI findings with analyses of gut microbiota composition, SCFA profiles, and systemic cytokine dynamics, this study explores the underlying mechanisms driving AD progression. Using 3 × Tg-AD mice at 6 months-old (6-MO) and 12 months-old (12-MO), it investigates how neuroinflammation and gut dysbiosis influence one another, ultimately shaping disease trajectory.

To further elucidate these interactions, DKI metrics are correlated with peripheral inflammatory markers and microbial changes, providing a comprehensive understanding of the brain-gut axis in AD progressive pathology at age of 6-MO and 12-MO. Behavioral assessments, such as the T-maze test, are used to evaluate working memory and decision-making abilities, linking these cognitive outcomes to neuroinflammation and gut dysbiosis. This multidimensional approach integrates noninvasive imaging, biochemical analyses, and behavioral testing, offering valuable insights into the mechanisms underlying AD progression and identifying potential biomarkers for early diagnosis and monitoring.

## Results and Discussion

### Working Memory Impairment in 3 × Tg-AD Mice during Disease Progression

This study explores the progressive working memory deficits in 3 × Tg-AD mice using the T-maze paradigm, a behavioral assay that effectively links cognitive performance to underlying neuropathology. Combining behavioral neuroscience and molecular biology, this study provides insights into AD-associated neurodegeneration. The T-maze task leverages the natural exploratory tendency of mice to alternate between goal arms, with the alternation rate serving as a measure of working memory accuracy.^26^

**Figure 1:**
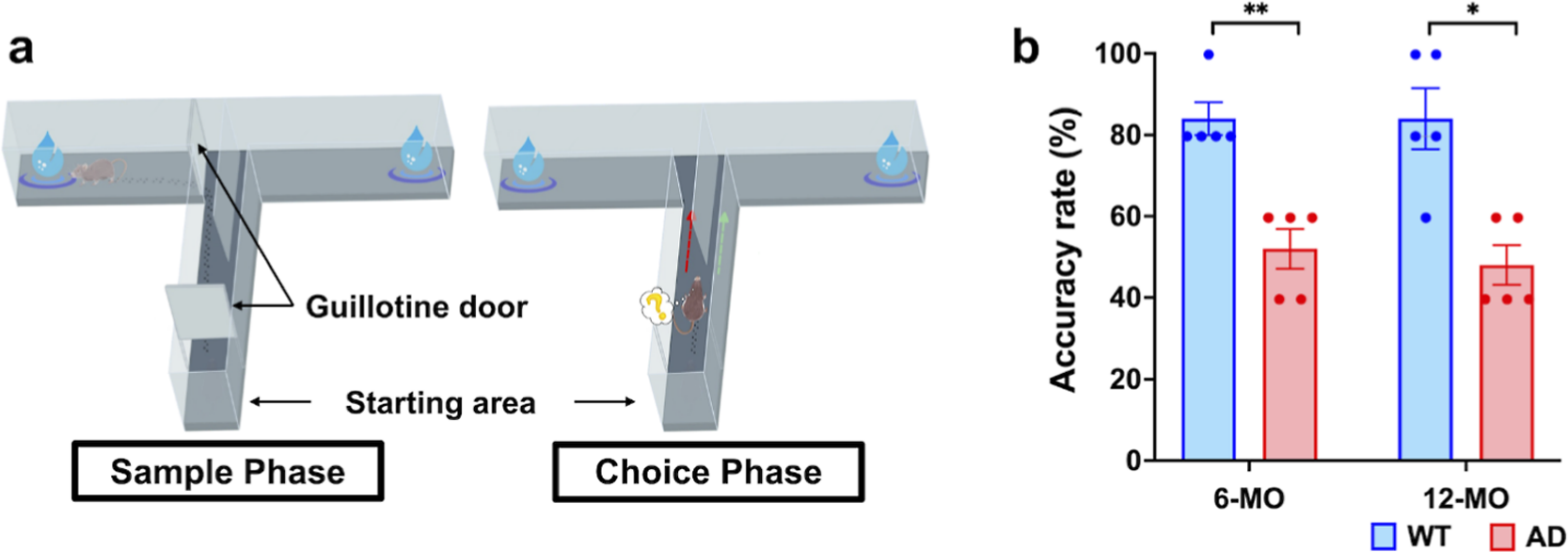
Assessment of working memory performance in AD mice using the T-maze task. (a) Schematic representation of the T-maze task conducted on experimental day 1 to assess working memory. The task involves two phases: the sample phase and the choice phase. In the sample phase, the mouse is placed in the starting area for 10 s with the guillotine door closed. Once the door is opened, the mouse explores the maze and selects one of two arms, where it remains for 30 s for exploration. In the choice phase, the mouse is returned to the starting area and makes a second selection. Choosing the opposite arm (green arrow) indicates intact working memory, while revisiting the same arm (red arrow) indicates memory deficits. (b) Accuracy rates in WT and AD mice at 6-MO and 12-MO. WT mice showed significantly higher accuracy at both time points, reflecting intact working memory, while AD mice displayed impaired performance, highlighting memory deficits. Statistical analysis (Mann–Whitney *U* test, FDR-adjusted): *p < 0.05, **p < 0.01. Data are shown as mean ± SEM, with individual points representing sample sizes.

As illustrated in Figure 1, 6-MO AD mice exhibited significantly reduced alternation accuracy (52% ± 10.95%) compared to age-matched WT group (84% ± 8.94%, **p < 0.01, Mann–Whitney test). Similarly, 12-MO AD mice showed significantly lower performance (48% ± 10.95%) relative to 12-MO WT mice (84% ± 16.73%, *p < 0.05, Mann–Whitney *U* test). Within the AD groups, a subtle yet consistent decline in memory performance was observed, with 12-MO AD mice showing poorer accuracy than 6-MO AD mice. These findings are consistent with previous studies, which have reported significant working memory impairments in 3 × Tg-AD mice compared to WT counterparts.^27,28^

The observed deficits likely stem from disruptions in the Papez circuit, a neural network comprising the HIPP, entorhinal cortex (EC), and fornix, which plays a pivotal role in spatial navigation and memory formation.^29^ Neurodegeneration in these regions, a hallmark of AD, may impair memory retrieval and alternation behaviors. Moreover, interconnected brain regions such as the medial prefrontal cortex (mPFC), nucleus accumbens (NAc), and striatum (STR), which regulate cognitive flexibility and decision-making, are also known to be affected in AD, further contributing to the observed impairments.^30,31^ The progressive decline in working memory performance with age aligns with the accumulation of pathological hallmarks, including Aβ plaques and tau tangles, which disrupt synaptic integrity and plasticity in key memory regions.^32^ Furthermore, neuroinflammation, characterized by elevated cytokine levels and activated glial cells, exacerbates neuronal dysfunction and network disintegration, accelerating cognitive decline in AD.^33^

In summary, the results of this study demonstrate a significant decline in working memory accuracy in 3 × Tg-AD mice compared to WT group, with increasing severity observed over time. These findings support the role of neuropathological changes in driving cognitive impairments and emphasize the utility of this model in studying the progression of AD.

### Neuroinflammatory and Microstructural Alterations in 3 × Tg-AD Mice Assessed by DKI

**Figure 2:**
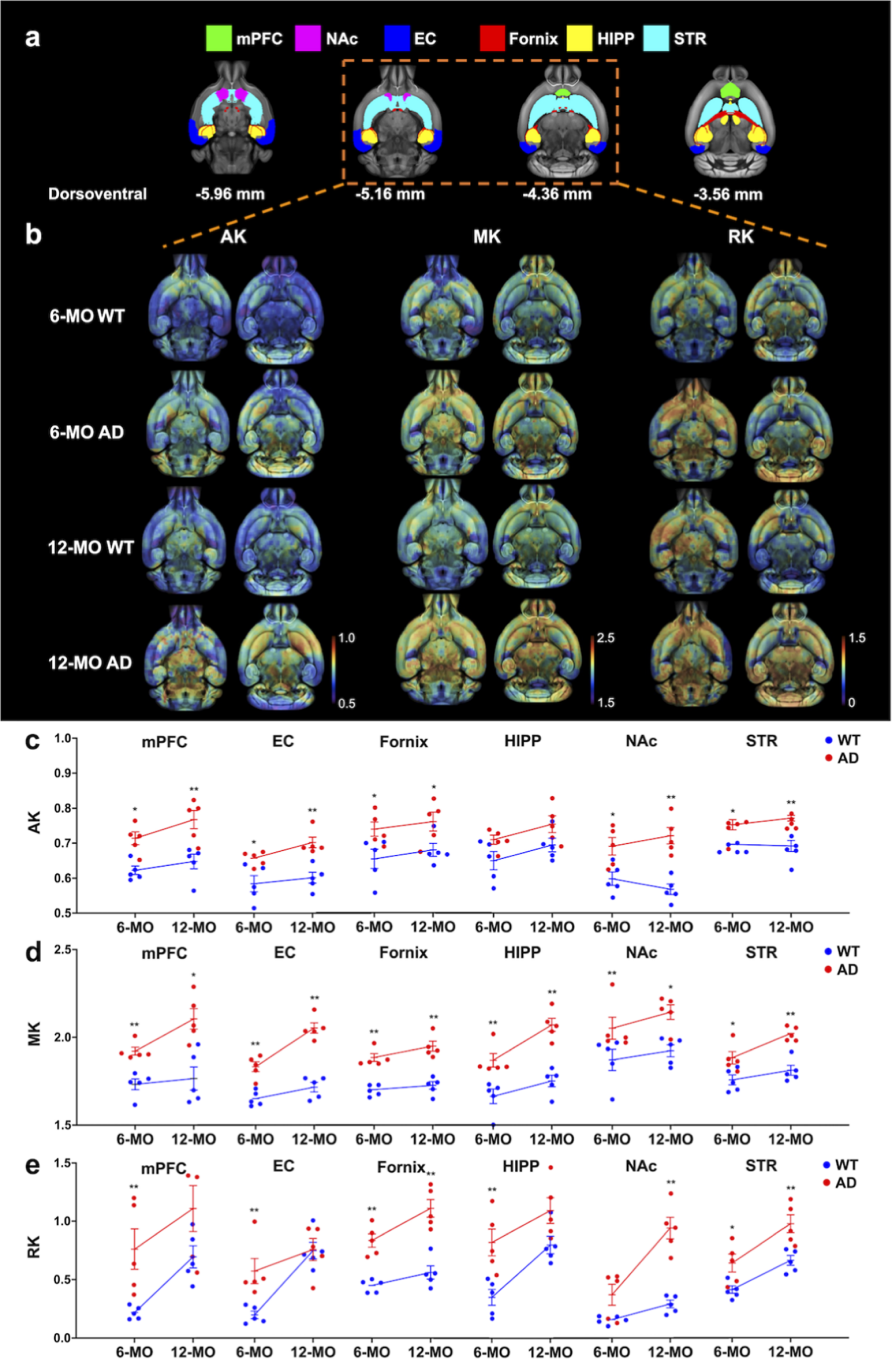
Brain microstructural changes in AD mice analyzed through DKI. (a) ROIs for DKI analysis, including the mPFC (green), EC (blue), fornix (red), HIPP (yellow), NAc (magenta), and STR (cyan), are shown overlaid on T2-weighted images at four dorsoventral coordinates (−5.96, −5.16, −4.36, and −3.56 mm). (b) Parametric maps for AK, MK, and RK are displayed for WT and AD mice at 6-MO and 12-MO. High values are represented in red, while low values are in purple, with color scales provided for AK (0.5–1.0), MK (1.5–2.5), and RK (0.0–1.5). AD mice show marked alterations in these parameters compared to WT mice. (c–e) Quantitative analysis of AK, MK, and RK values across ROIs at 6-MO and 12-MO. (c) AK values progressively increase in AD mice relative to WT, with significant differences (*p < 0.05, **p < 0.01, Mann–Whitney *U* test, FDR-adjusted) observed across all ROIs. (d) MK values are consistently higher in AD mice, particularly at 12-MO, highlighting microstructural disruptions, with statistical significance indicated (*p < 0.05, **p < 0.01). (e) RK values show similar trends, with significant increases in AD mice, most prominently in the HIPP and STR at 12-MO (*p < 0.05, **p < 0.01). Data are shown as mean ± SEM, with individual data points representing sample sizes. These findings underscore age- and region-specific microstructural changes in AD mice, with DKI metrics (AK, MK, and RK) serving as sensitive indicators of neurodegeneration.

To evaluate disease-related microstructural changes in 3 × Tg-AD mice, DKI was employed to analyze AK, MK, and RK. These parameters are sensitive biomarkers of tissue integrity and neuroinflammation. Six ROIs related to memory processing and the Papez circuit—mPFC, NAc, EC, fornix, HIPP, STR—were analyzed in both 6-MO and 12-MO cohorts (Figure 2a). The results highlighted significant neuroinflammatory and structural abnormalities in AD mice compared to WT controls across all age groups (Figure 2b–e).

For AK, the 6-MO AD group exhibited significantly higher values than the 6-MO WT group in the mPFC (*p < 0.05, Mann–Whitney *U* test) and the NAc, EC, fornix, and STR (*p < 0.05, Mann–Whitney *U* test), while no differences were observed in the HIPP. Similarly, in the 12-MO cohort, AK values were significantly elevated in the AD group compared to the WT group in the mPFC, NAc, EC, STR (**p < 0.01, Mann–Whitney *U* test) and fornix (*p < 0.05, Mann–Whitney *U* test), with no changes in the HIPP (Figure 2c).

For MK, the 6-MO AD group showed significantly higher values across all ROIs, including the mPFC, NAc, EC, fornix, HIPP (**p < 0.01, Mann–Whitney test), and STR (*p < 0.05, Mann–Whitney *U* test). Similarly, in the 12-MO cohort, MK values were significantly elevated in all six ROIs in the AD group, with mPFC and NAc (*p < 0.05, Mann–Whitney *U* test) and EC, fornix, HIPP, and STR (**p < 0.01, Mann–Whitney *U* test) showing marked increases (Figure 2d).

For RK, the 6-MO AD group exhibited significantly higher values compared to the WT group in the mPFC, EC, fornix, HIPP (**p < 0.01, Mann–Whitney *U* test), and STR (*p < 0.05, Mann–Whitney *U* test), but no significant differences were observed in the NAc. In the 12-MO cohort, RK was significantly elevated in the fornix, NAc, and STR (**p < 0.01, Mann–Whitney *U* test) in the AD group compared to the WT group, while other ROIs showed no significant differences (Figure 2e). These findings suggest that elevated DKI metrics are indicative of neuroinflammation and amyloid-related structural changes in AD. Increased AK, MK, and RK values in AD mice, particularly in regions of the Papez circuit, align with prior studies showing that Aβ deposition and glial activation restrict water diffusion, leading to elevated kurtosis values.^34−36^ MK and RK, in particular, have been shown to be highly sensitive to pathological changes in brain tissue, reflecting both amyloid deposition and gliosis in memory-related regions.^37^

The absence of significant changes in HIPP AK values, despite alterations in other ROIs, may reflect regional variability in pathological progression. However, the consistent increases in MK and RK across most ROIs further emphasize the role of neuroinflammation and amyloid pathology in driving these structural changes. These findings reinforce the utility of DKI parameters as robust indicators of microstructural abnormalities and neuroinflammation in AD,^38^ providing valuable insights into the underlying mechanisms of memory impairment. By linking elevated AK, MK, amd RK values to neuroinflammatory and neurodegenerative changes, DKI metrics offer biomarkers for tracking progression in AD.

To further explore connectively disruptions, we performed white matter tractography in 3 × Tg-AD mice, with detailed results provided in the Supporting Information (Figure S1 in Supporting Information Note 1). FA heatmaps were generated for the WT and AD groups at 6-MO and 12-MO (Figure S1b). The WT group displayed preserved white matter integrity, while the AD group exhibited widespread FA reductions, particularly in the HIPP–mPFC, hipp–EC, and mPFC–Fornix tracts. These reductions were more pronounced at 12-MO, indicating a progressive decline in white matter integrity over time (Figure S1c).

Our findings demonstrate that neuroinflammation induces widespread microstructural disruptions, particularly in white matter pathways connecting the mPFC, HIPP, and EC. Notably, HIPP_EC and mPFC–STR tracts, which showed minimal FA changes at 6-MO, exhibited significant reductions at 12-MO, suggesting progressive deterioration with chronic inflammation. This pattern supports the chronic nature of neuroinflammation, wherein sustained glial activation and oxidative stress contribute to ongoing axonal and myelin damage.^39^ These tractography results are consistent with the DKI findings, where elevated AK, MK, and RK values highlighted neuroinflammation and amyloid-related structural changes.^40^ The combined reduction in FA and increased kurtosis metrics suggest that neuroinflammation disrupts both local microstructure and global white matter connectivity.

### Neuroinflammatory Variations in 3 × Tg-AD Mice: a Cell Composition Perspective

**Figure 3:**
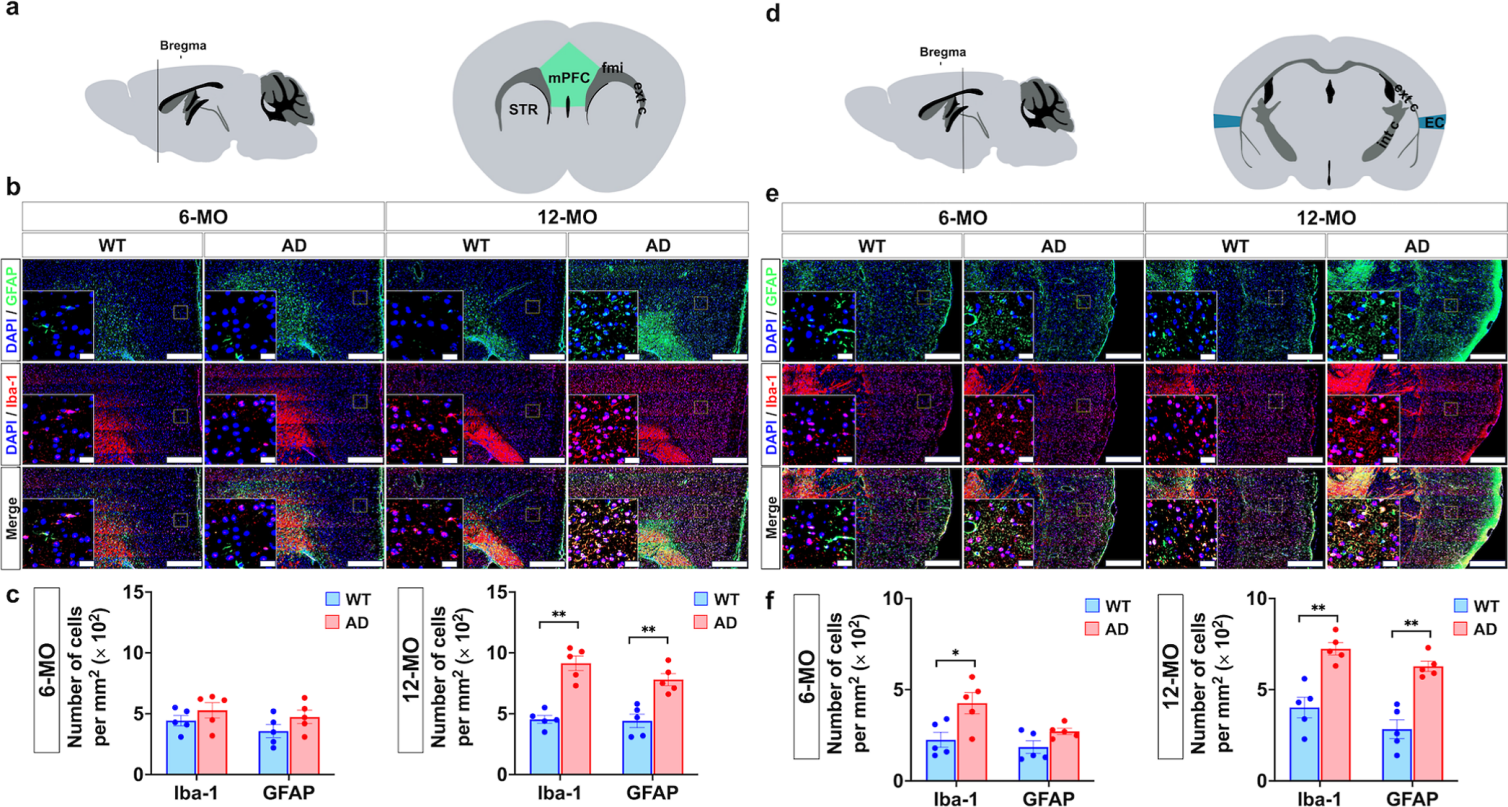
IHC analysis for Iba-1 and GFAP in the mPFC and EC. (a) Left panel: schematic sagittal section of the mouse brain. Right panel: coronal view highlighting the mPFC (green). (b) Representative IHC images of mPFC sections from 6-MO and 12-MO WT and AD mice. Sections were stained with DAPI (blue) for nuclei, Iba1 (red) for microglia, and GFAP (green) for astrocytes. Insets magnify boxed regions to detail cellular morphology. Scale bars: 400 μm (main images) and 40 μm (insets). Iba1- and GFAP-positive cells were quantified in 10 randomly selected mPFC regions per mouse (c). In comparison to WT controls, Iba1 and GFAP expression levels were significantly elevated in AD mice 12-MO (*p < 0.05, **p < 0.01; Mann–Whitney *U* test, FDR-adjusted). Although a slight increase in Iba1 and GFAP expression was observed in 6-MO AD mice, the difference was not statistically significant compared to age-matched WT mice. (d) Left panel: Schematic sagittal section of the mouse brain. Right panel: coronal view highlighting the EC (blue). (e) Representative IHC images of EC sections stained as in (b). Insets magnify boxed regions, emphasizing glial activation. Scale bars: 400 μm (main images) and 40 μm (insets). (f) Quantification of Iba1 and GFAP-positive cells, analyzed similarly to (c), revealed significantly increased Iba1 expression in AD mice at both time points (*p < 0.05, **p < 0.01; Mann–Whitney *U* test, FDR-adjusted). Additionally, GFAP expression in astrocytes was significantly elevated in 12-MO-old AD mice (**p < 0.01; Mann–Whitney *U* test, FDR-adjusted). Data are presented as mean ± SEM, with individual points representing measurements from different mice.

**Figure 4:**
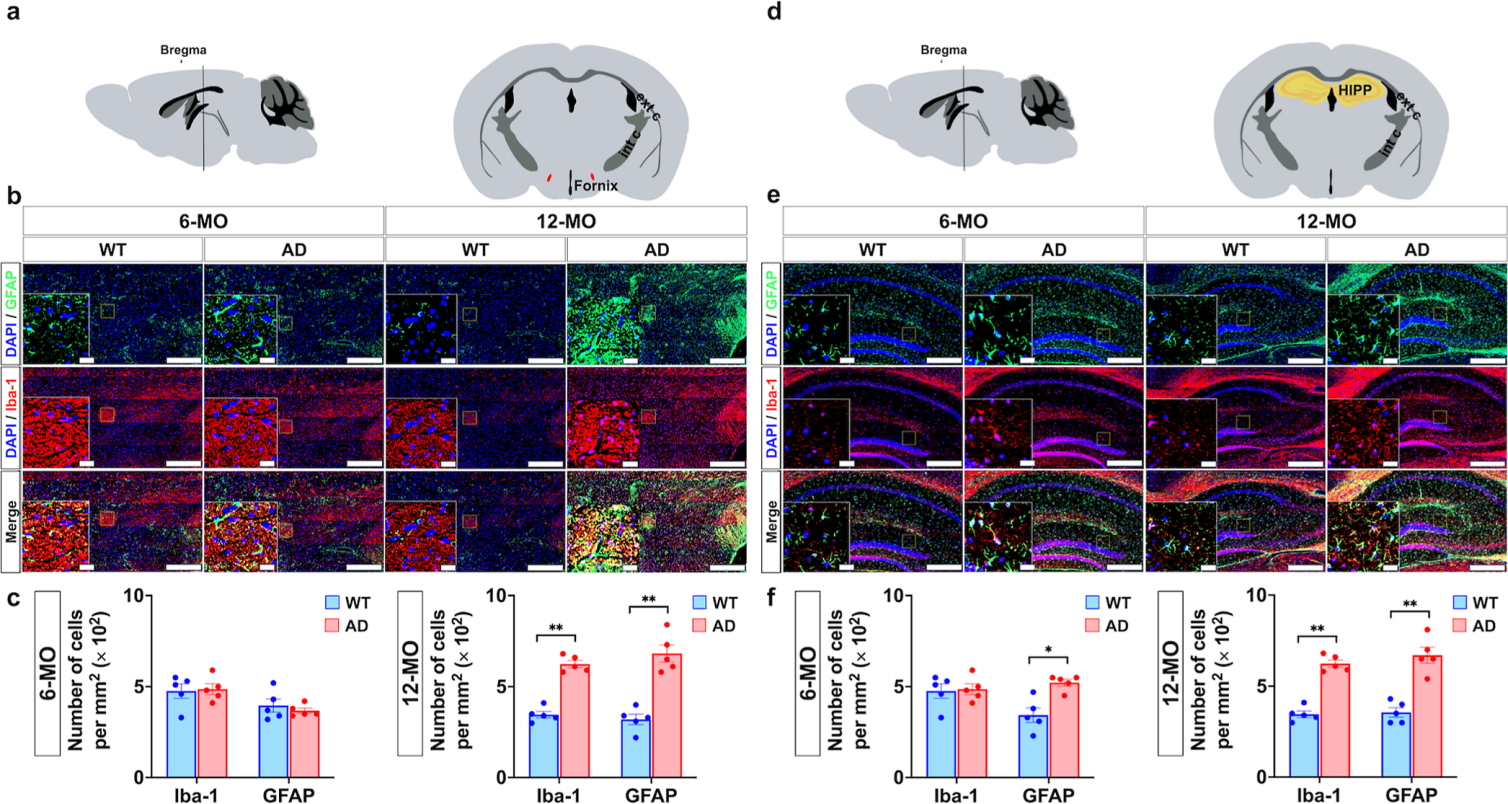
IHC analysis for Iba-1 and GFAP in the fornix and HIPP. (a) Left panel: schematic sagittal view of the mouse brain. Right panel: coronal section highlighting the fornix (red). (b) Representative IHC images of the fornix from 6-MO and 12-MO-old WT and AD mice. Sections were stained with DAPI (blue) for nuclei, Iba1 (red) for microglia, and GFAP (green) for astrocytes. Insets provide higher magnification of boxed regions, illustrating detailed cellular morphology. Scale bars: 400 μm (main images) and 40 μm (insets). (c) Quantitative analysis of Iba1- and GFAP-positive cells in the fornix, based on ten randomly selected areas per mouse, showed that only 12-MO-old AD mice exhibited significantly higher activation of microglia and astrocyte cells compared to age-matched WT controls. The significant increase in positive cell expression (**p < 0.01) was determined using the FDR-adjusted Mann–Whitney *U* test. (d) Left panel: schematic sagittal view of the mouse brain. Right: coronal section highlighting the HIPP (yellow). (e) Representative IHC images of the HIPP from 6-MO and 12-MO WT and AD mice, stained with DAPI, Iba1, and GFAP. Insets show magnified views of boxed regions, emphasizing glial activation. Scale bars: 400 μm (main images) and 40 μm (insets). (f) Quantitative analysis of GFAP-positive cells in the HIPP indicates significantly elevated astrocytes activation in AD mice compared to age-matched WT controls at both 6-MO (*p < 0.05) and 12-MO (**p < 0.01), using the Mann–Whitney *U* test with FDR adjustment. Additionally, Iba1 expression in microglia only was significantly elevated in 12-MO-old AD mice (**p < 0.01; Mann–Whitney *U* test, FDR-adjusted). Data are presented as mean ± SEM, with individual points representing measurements from different mice.

**Figure 5:**
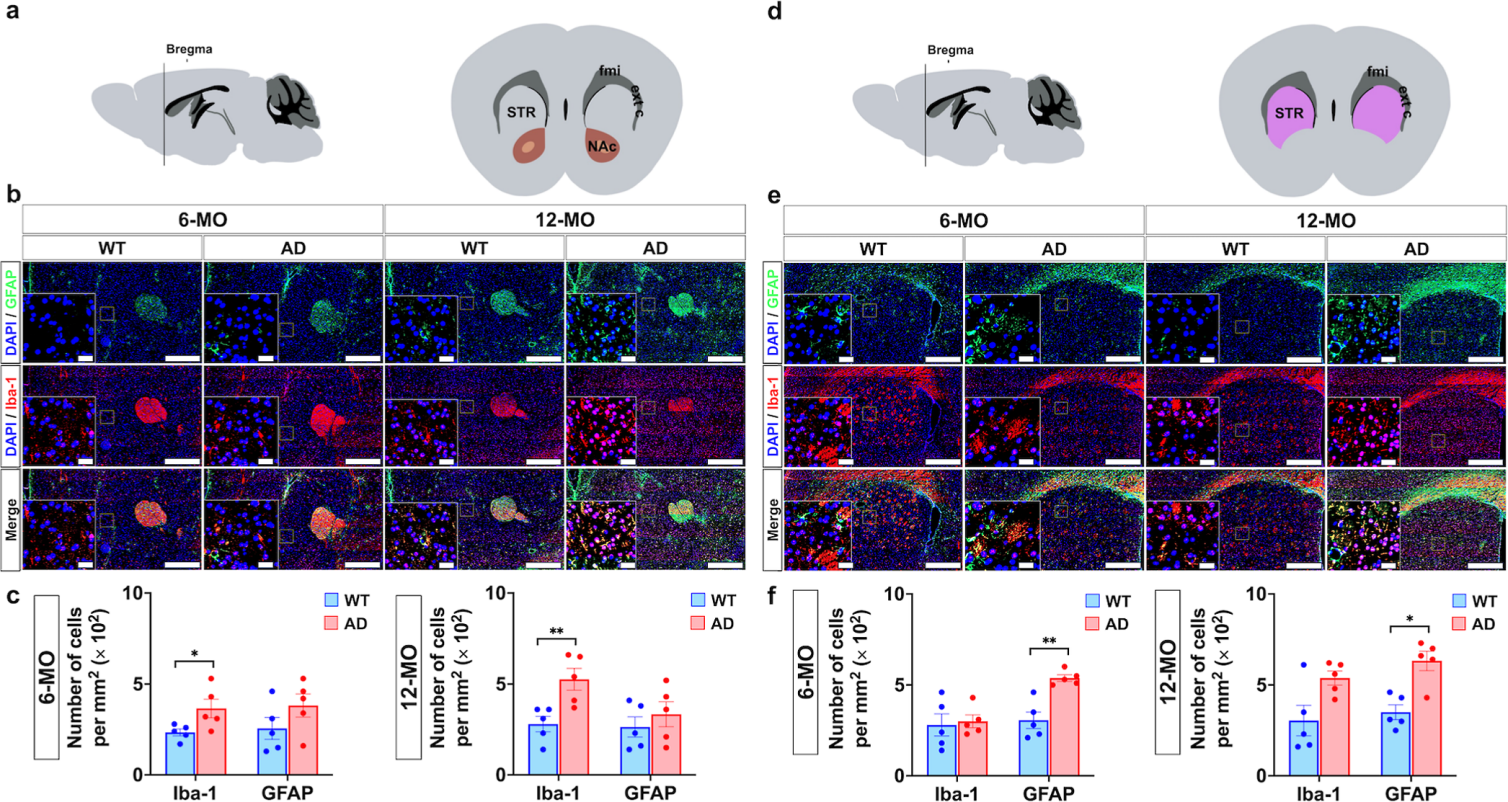
IHC analysis for Iba-1 and GFAP in the NAc and STR. (a) Left panel: schematic sagittal view of the mouse brain. Right panel: coronal section highlighting the NAc (red). (b) Representative IHC images of NAc sections from 6-MO and 12-MO-old WT and AD mice. Sections were stained with DAPI (blue) to label nuclei, Iba1 (red) to identify microglia, and GFAP (green) to mark astrocytes. Insets show higher magnification of boxed regions for detailed cellular morphology. Scale bars: 400 μm (main images) and 40 μm (insets). (c) Quantitative analysis of Iba1 expression in the NAc, based on 10 randomly selected regions per mouse, revealed significantly higher Iba1 levels in AD mice at both 6-MO (*p < 0.05) and 12-MO (**p < 0.01), as determined by the Mann–Whitney *U* test with FDR adjustment. While 6-MO and 12-MO AD mice exhibited increased trends in GFAP-positive astrocytes, no significant difference in GFAP expression was observed when compared to the WT group. (d) Left panel: schematic sagittal view of the mouse brain. Right: coronal section highlighting the STR (purple). (e) Representative IHC images of STR sections, stained as in (b). Insets provide magnified views of boxed regions, emphasizing cellular morphology. Scale bars: 400 μm (main images) and 40 μm (insets). (f) Quantitative analysis of Iba1 and GFAP-positive cells in the STR revealed significantly increased GFAP protein expression in astrocytes of AD mice compared to WT controls at both 6-MO (*p < 0.05) and 12-MO (**p < 0.01), as determined by the Mann–Whitney *U* test with FDR adjustment. Although 6-MO and 12-MO AD mice exhibited higher Iba1 levels, no significant differences in microglial activation were observed compared to the WT group. Data are presented as mean ± SEM, with individual points reflecting measurements from different mice.

Neuroinflammation, characterized by microgliosis and astrogliosis, is a hallmark of AD pathology. Microgliosis reflects microglial activation, often contributing to phagocytosis and neuroinflammatory signaling, while astrogliosis indicates astrocyte activation, frequently associated with scar formation and chronic inflammation. To quantify neuroinflammation, we assessed microglial and astrocytic activation using immunohistochemical (IHC) staining for Iba-1 and glial fibrillary acidic protein (GFAP), respectively. The spatial and temporal patterns of Iba-1 and GFAP expression were examined across six ROIs: mPFC and EC (Figure 3), fornix and HIPP (Figure 4), NAc and STR—were examined for Iba-1 and GFAP expression patterns (Figure 5).

At 6-MO, the AD group showed significantly elevated expression in the EC (*p < 0.05, Mann–Whitney *U* test) and NAc (*p < 0.05, Mann–Whitney *U* test) compared to the WT group. By 12-MO, Iba-1 levels were significantly elevated in the AD group across five ROIs—mPFC (**p < 0.01, Mann–Whitney *U* test), NAc, EC, fornix, and HIPP (**p < 0.01, Mann–Whitney *U* test)—with no significant changes in the STR. For GFAP, 6-MO AD mice exhibited significantly higher expression levels in the HIPP (*p < 0.05, Mann–Whitney *U* test) and STR (**p < 0.01, Mann–Whitney *U* test) compared to WT controls. In the 12-MO cohort, GFAP expression was significantly elevated in five ROIs—mPFC, EC, fornix, HIPP (**p < 0.01, Mann–Whitney *U* test), and STR (*p < 0.05, Mann–Whitney *U* test)—with no significant changes observed in the NAc. These data suggest progressive neuroinflammation in regions critical for memory and cognitive function as the disease advances.

Our findings reveal a progression increase in neuroinflammation in 3 × Tg-AD mice, with microglial and astrocytic activation intensifying in memory-related brain regions as the disease advances from 6-MO to 12-MO of age. Notably, while prior studies have highlighted early microglial activation around Aβ plaques,^41,42^ our study provides a detailed, region-specific analysis of both microgliosis and astrogliosis. These observations highlight the differential vulnerability of brain regions involved in the Papez circuit and memory processing, including the HIPP, EC, and fornix.^43^ The prominent elevation of GFAP in the HIPP and STR at 6-MO suggests that astrocytic responses may precede or amplify microglial activation in certain regions, a pattern also observed in human AD biopsy samples.^44−47^ The lack of significant changes in certain regions, like the STR and NAc, suggests a complex relationship between region-specific vulnerabilities, amyloid deposition, and neuroinflammatory progression.^48^

### Variations in Inflammatory Cytokines in 3 × Tg-AD Mice: Implications for AD Pathogenesis

**Figure 6:**
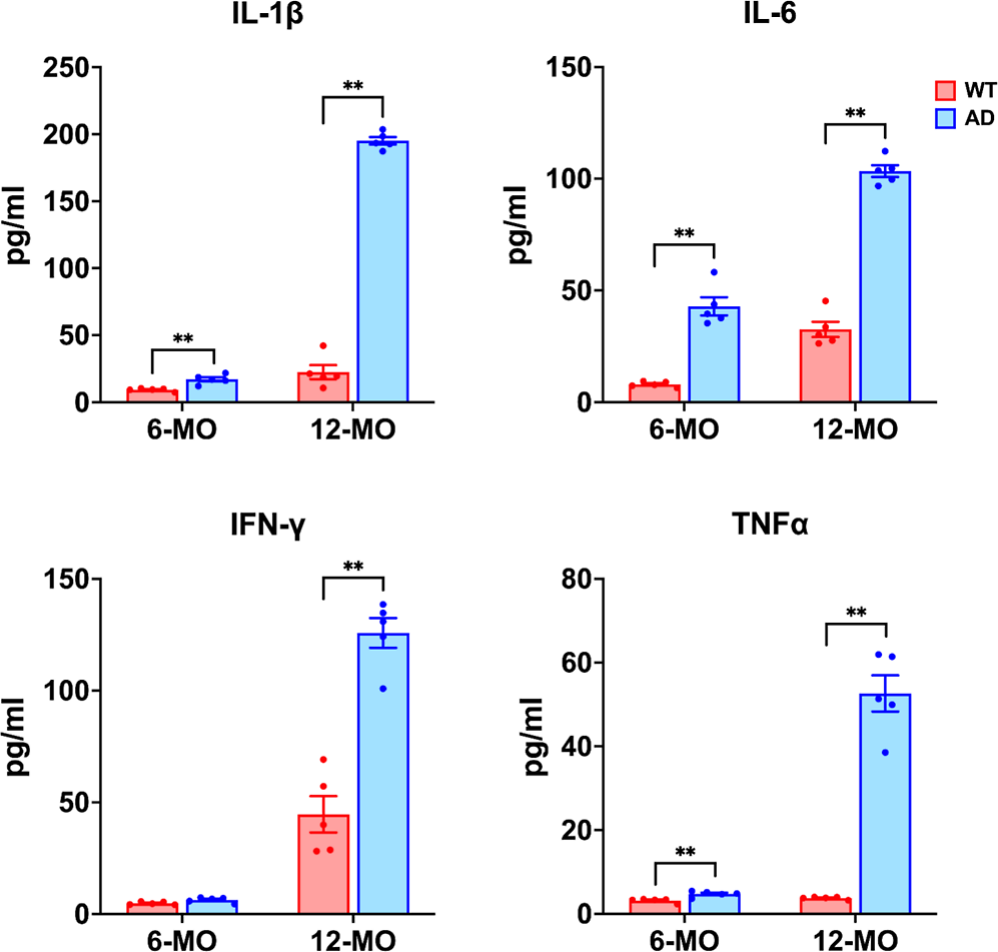
Comparison of pro-inflammatory cytokine levels between WT and AD mice at 6 and 12-MO. Plasma levels of pro-inflammatory cytokines, including IL-1β, IL-6, IFN-γ, and TNFα, were measured in WT and AD mice at 6-MO and 12-MO. IL-1β: levels were significantly higher in AD mice compared to WT controls at both 6-MO and 12-MO (**p < 0.01, FDR-adjusted Mann–Whitney *U* test). IL-6: AD mice exhibited markedly elevated IL-6 levels compared to WT mice at both time points (**p < 0.01). IFN-γ: a significant increase in IFN-γ levels was observed in AD mice at 12-MO (**p < 0.01), with minor changes at 6-MO. TNFα: TNF-α levels were significantly elevated in AD mice at 12-MO (**p < 0.01), with differences at 6-MO. Data are presented as mean ± SEM, with individual data points shown for each group.

Pro-inflammatory cytokines such as IFN-γ, IL-1β, IL-6, and TNFα play critical roles in AD progression, driving synaptic dysfunction, neuronal death, and cognitive decline. Serum levels of these cytokines were analyzed in 3 × Tg-AD mice at 6-MO and 12-MO, revealing distinct patterns of expression during disease progression (Figure 6).

At 6-MO, the AD group exhibited a higher IFN-γ level compared to WT controls, though this difference was not statistically significant. By 12-MO, however, IFN-γ levels in the AD group were significantly elevated compared to WT controls (**p < 0.01, Mann–Whitney *U* test). IFN-γ facilitates microglial phagocytosis of Aβ but also exacerbates inflammation by promoting leukocyte infiltration and increasing neuronal damage.^49,50^ Elevated IFN-γ levels also promote the expression of cell adhesion molecules and chemokines, facilitating T-cell migration and amplifying neuronal damage, especially in the presence of extracellular Aβ deposition.^51,52^ This dual role underscores IFN-γ′s complexity in AD pathogenesis.

Similarly, Both the 6-MO and 12-MO AD groups showed significantly higher IL-1β levels compared to WT controls (**p < 0.01, Mann–Whitney *U* test), highlighting its role in amplifying Aβ accumulation and BBB permeability, further exacerbating Aβ accumulation, neuroinflammation and cognitive decline.^53^

IL-6 levels were significantly elevated in both 6-MO and 12-MO AD mice compared to WT controls (**p < 0.01, Mann–Whitney *U* test). Reflecting its dual role in neuroinflammation and tau hyperphosphorylation via the activation of JAK/STAT, NMDA receptor, and MAPK-p38 pathways.^54,55^ Elevated IL-6 levels can also activate neuronal NADPH oxidase, leading to oxidative stress and impairments in spatial learning and memory.^56,57^

Likewise, TNFα levels were elevated in both cohorts (**p < 0.01, Mann–Whitney *U* test), reinforcing its contribution to Aβ accumulation, tau pathology, and chronic neuroinflammation.^58^ Sustained TNFα elevation leads to neuronal cell death and synaptic dysfunction, directly contributing to memory loss and learning deficits in AD.^59^

Our findings demonstrate that IFN-γ, IL-1β, IL-6, and TNFα collectively facilitate various pathological mechanisms in AD, including Aβ clearance impairment, neuroinflammation, and tau pathology.^60^ Their elevated levels disrupt synaptic integrity and neurogenesis, further accelerating cognitive decline.^61^ These findings emphasize the critical role of inflammatory cytokines in AD pathogenesis and their potential as therapeutic targets.

### Correspondence of DKI Metrics and Neuroinflammation

DKI provides insights into microstructural complexity, capturing alterations in tissue architecture caused by Aβ deposition and neuroinflammation.^22^ MK, reflecting overall diffusion restriction, increases due to reactive gliosis, extracellular matrix remodeling, and neuronal loss, leading to heightened tissue heterogeneity.^62^ RK, measuring diffusion perpendicular to white matter tracts, is particularly sensitive to myelin integrity and astrocyte proliferation, making it a reliable marker of demyelination and gliosis.^63^ AK, which reflects diffusion along the principal fiber direction, rises in response to axonal swelling, cytoskeletal breakdown, and dystrophic neurite formation, indicating neuronal damage associated with Aβ pathology.^64^ Mechanistically, Aβ plaques serve as dense extracellular barriers that hinder water diffusion, leading to elevated MK and RK.^65^ Additionally, gliosis further restricts water mobility by increasing microstructural complexity. Notably, MK and RK are strongly associated with neuroinflammation, with higher values reflecting the structural alterations caused by activated glial cells.^66^ Neuroinflammatory pathways amplify these effects, further increasing cellular density and swelling, thereby reinforcing DKI metric elevations associated with AD neuroinflammation.^67^

Conversely, AK alterations may reflect disruptions in axonal integrity and neurodegeneration, further emphasizing the role of DKI in detecting pathological changes.^68^ Experimental studies in AD mouse models strongly correlate increased MK, RK, and AK with histological markers of neuroinflammation, and neuronal damage, with MK and AK rising early in the progression of AD.^25,69^ In human studies, DKI abnormalities are detected even in cognitively normal individuals with amyloid positivity, suggesting that DKI metrics serve as early biomarkers of AD pathology.^36^ Furthermore, progressive alterations in MK and RK correlate with worsening cognitive function, reinforcing DKI’s potential for tracking disease progression and assessing therapeutic interventions.^20^

Clinically, DKI is a sensitive imaging biomarker for early diagnosis, longitudinal monitoring, and treatment assessment in AD.^70^ Serial DKI scans can quantitatively track disease progression by measuring MK, RK, and AK changes over time, providing an objective measure of ongoing neuroinflammatory and neurodegenerative processes.^71^ Importantly, DKI metrics offer a means to evaluate therapies aimed at reducing neuroinflammation or Aβ accumulation, with successful anti-inflammatory or antiamyloid treatments expected to normalize kurtosis values, an outcome DKI can capture in longitudinal follow-ups.^25^ This ability to detect subtle yet clinically significant microstructural changes make DKI a valuable tool for both understanding AD pathology and optimizing treatment strategies. The progressive nature of DKI metric alterations in AD highlights their clinical relevance in assessing neuroinflammation-driven neurodegeneration, reinforcing DKI as a crucial modality for early detection, disease monitoring, and therapeutic evaluation in neurodegenerative disorders.^25^

### Disease-Related Variations in Gut Microbiota Composition and Diversity in 3 × Tg-AD Mice

**Figure 7:**
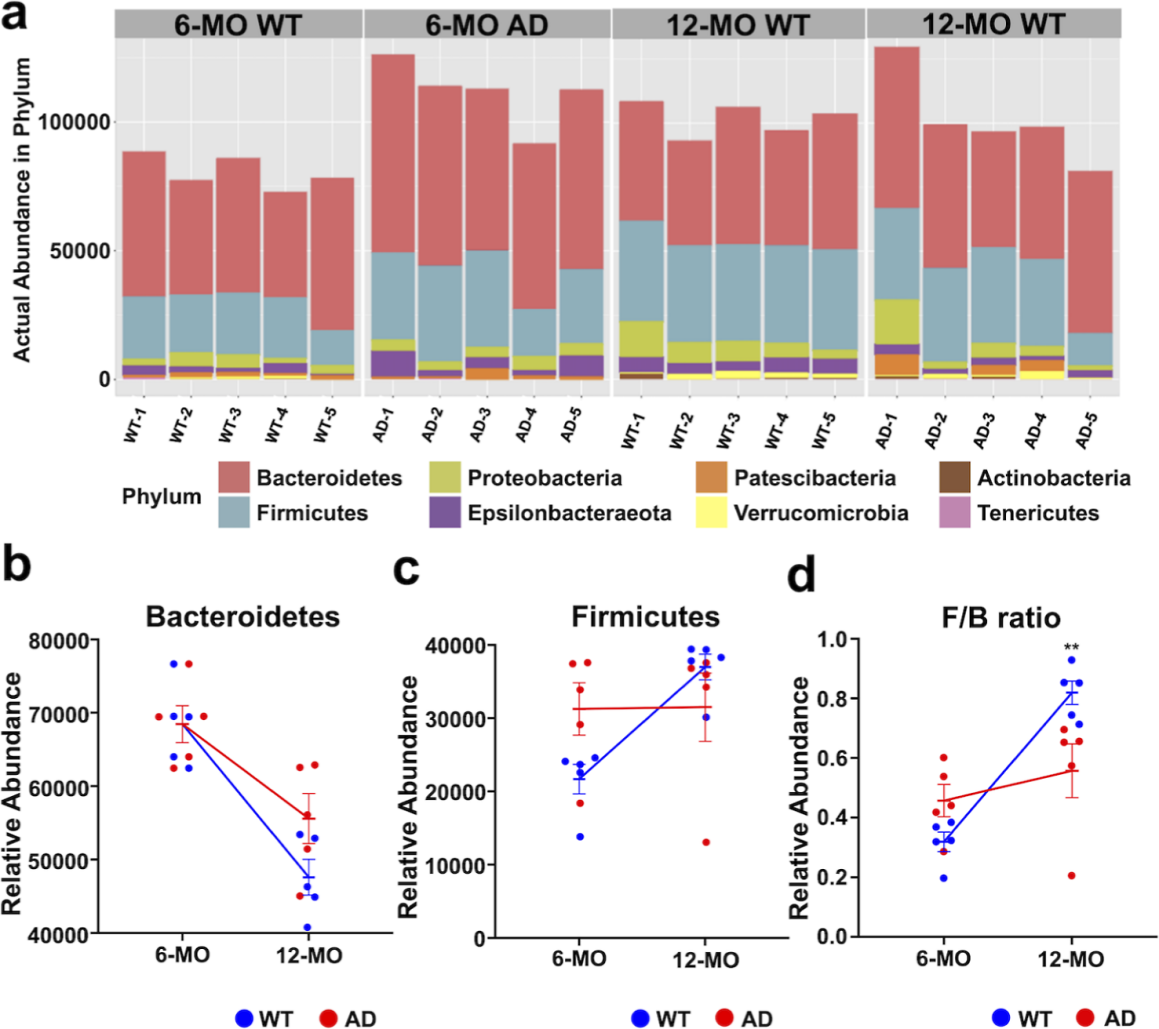
Alterations in microbiota abundance in AD mice. (a) Stacked bar plots showing the actual abundance of microbiota at the phylum level across 6-MO and 12-MO WT and AD mice. The dominant phyla include *Bacteroidetes* (red) and *Firmicutes* (blue), followed by *Proteobacteria*, *Verrucomicrobia*, and others. A reduction in *Bacteroidetes* is observed in AD mice over time, while *Firmicutes* remain consistently lower in AD mice compared to WT at 12-MO, indicating distinct microbiota alterations associated with the AD condition. (b) Relative abundance of *Bacteroidetes* declines over time in both WT and AD groups. However, there is no statistical significance between groups. (c) Relative abundance of *Firmicutes* appears to increase slightly in WT mice at 12-MO, whereas AD mice exhibit a decline, suggesting distinct microbial dynamics between the groups with no statistical significance. (d) The *F*/*B* ratio, an indicator of gut microbial composition, is significantly elevated in WT mice at 12-MO, while AD mice show a more modest increase (**p < 0.01, FDR-adjusted Mann–Whitney *U* test), suggesting a different trajectory in microbiota changes between groups. Data are presented as mean ± SEM, with individual data points shown.

**Figure 8:**
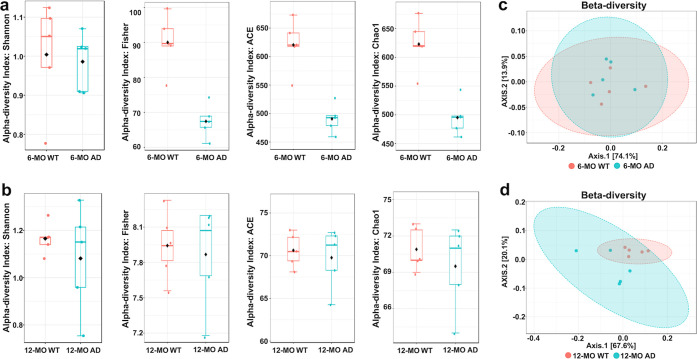
Microbiota diversity analysis in AD mice. (a) Alpha-diversity indices [Shannon, Fisher, abundance-based coverage estimator (ACE), and Chao1] were used to assess microbial richness and diversity in 6-MO WT and AD mice. AD mice exhibit a slightly lower alpha diversity across some indices, but the differences are not statistically significant, indicating a potential trend toward reduced microbial richness and evenness without substantial alterations at this stage. Black dots within the box plots represent group means. (b) At 12-MO, alpha diversity does not show a consistent trend between AD and WT groups. While ACE and Chao1 indices remain slightly lower in AD mice, Shannon and Fisher indices show overlapping distributions, suggesting that microbial diversity is relatively stable over time. Black dots within the box plots represent group means. (c) Beta-diversity analysis based on PCoA of 6-MO groups shows partial overlap between WT and AD mice, indicating minor compositional differences in gut microbiota. (d) Beta-diversity analysis of 12-MO groups reveals a greater separation between WT and AD mice, suggesting a more pronounced shift in microbiota composition over time.

Gut microbiota dysbiosis has emerged as a key contributor to AD pathogenesis, affecting systemic inflammation and neuroinflammation via the gut-brain axis. In this study, we assessed the relative abundance of major bacterial phyla, the *F*/*B* ratio, and alpha and beta diversity in 3 × Tg-AD and WT mice at age of 6-OM and 12-MO (Figures 7 and 8).

Analysis of relative phylum abundance (Figure 7a) revealed that *Bacteroidetes* and *Firmicutes* were dominant across all groups, consistent with previous findings (52). At 6-MO, the relative abundance of *Bacteroidetes* and *Firmicutes* was comparable between AD and WT groups (Figure 7b,c). By 12-MO, both groups exhibited changes in microbial composition over time. While *Bacteroidetes* showed a decreasing trend in both AD and WT mice, there were no statistically significant differences between groups. Similarly, Firmicutes showed a trend toward an increase in WT mice and a decline in AD mice, but these changes were also not statistically significant. Although these differences were not statistically significant, they suggest distinct microbial shifts in AD mice during disease progression, potentially contributing to gut microbiota alterations associated with advanced AD. Despite these trends, the *F*/*B* ratio exhibited a more pronounced shift, with WT mice showing an increase at 12-MO, while AD mice demonstrated a more modest change These shifts resulted in a significantly lower *F*/*B* ratio in the 12-MO AD group compared to WT (**p < 0.01, Mann–Whitney *U* test) (Figure 7d). The reduction in the *F*/*B* ratio, linked to increased gut permeability, systemic inflammation, and impaired SCFA production, highlights a dysregulated gut microbial ecosystem in AD progression.^52,53^

Alpha diversity, reflecting species richness and evenness, was assessed using Shannon, Fisher, ACE, and Chao1 indices. The Shannon index, which considers both species richness and evenness, showed only minor differences between AD and WT mice at 6 and 12-MO, suggesting that the overall bacterial balance remained stable despite other diversity shifts (Figure 8a,b). Similarly, Fisher’s index, a robust estimator of species richness, showed no significant differences, indicating that dominant microbial populations were largely preserved across groups. In contrast, the ACE and Chao1 indices, which emphasize rare species richness, were slightly lower in AD mice at both time points. This suggests a potential loss of low-abundance bacterial taxa associated with disease progression. At 6-MO, the AD group exhibited a slight but statistically insignificant reduction in alpha diversity compared to WT controls, indicating an early but subtle trend of reduced microbial richness. By 12-MO, alpha diversity patterns remained inconsistent, with some indices suggesting stability or compensatory changes (Figure 8a,b).

Beta diversity based on principle coordinate analysis (PCoA), which assesses microbial community structure, showed no significant separation between 6-MO AD and WT groups (Figure 8c), indicating early stage stability. However, at 12-MO, the AD group exhibited distinct clustering compared to WT controls (*r* = 0.472, **p < 0.01) (Figure 8d), reflecting substantial shifts in microbial composition during advanced disease stages. These findings highlight the progressive nature of gut dysbiosis in AD and its potential role in disease pathology.

The results demonstrate a clear progression in gut microbiota dysbiosis in 3 × Tg-AD mice, with advanced stages of AD characterized by significant shifts in microbial composition and diversity. The observed changes in the *F*/*B* ratio, relative abundance of bacterial phyla, and beta diversity provide critical insights into the role of gut microbiota in AD pathogenesis. At 6-MO, the lack of significant differences in the *F*/*B* ratio and beta diversity suggests that gut dysbiosis is still in its early stages. The slightly lower alpha diversity in the AD group reflects a potential trend toward reduced microbial richness and evenness. However, the absence of significant changes may indicate that compensatory mechanisms or the relatively mild impact of early neuroinflammatory processes have not yet led to substantial alterations in the gut microbiota. This aligns with previous findings that dysbiosis intensifies as AD pathology progresses, particularly during the transition from early to advanced disease stages.^72,73^ By 12-MO, significant differences in the *F*/*B* ratio and beta diversity between AD and WT groups underscore the advanced dysbiosis in AD mice. The reduced *F*/*B* ratio, driven by increased *Bacteroidetes* and decreased *Firmicutes*, is consistent with prior studies linking such microbial imbalances to gut barrier dysfunction, systemic inflammation, and impaired SCFA production.^19,74−76^ A lower *F*/*B* ratio has been associated with increased gut permeability, facilitating the translocation of microbial metabolites and inflammatory molecules into circulation, thereby exacerbating systemic inflammation and neuroinflammatory processes in the brain.^77,78^ This mechanism is further supported by the increased beta diversity in the 12-MO AD group, reflecting significant disruptions in microbial community structure, which likely contribute to disease progression through the gut-brain axis. The stability of alpha diversity at 12-MO suggests that changes in microbial community composition (as reflected in beta diversity) may play a more prominent role in AD progression than overall species richness or evenness. This finding highlights the importance of examining beta diversity in identifying microbial community-level changes that may not be apparent from alpha diversity metrics alone. The observed increase in *Bacteroidetes* and decrease in *Firmicutes* in the 12-MO AD group align with known microbial shifts in AD models and human patients.^79^*Bacteroidetes* are associated with pro-inflammatory responses, while *Firmicutes* are linked to SCFA production, which supports gut barrier integrity and exerts anti-inflammatory effects.^80−82^ The imbalance between these phyla in the AD group may drive systemic inflammation, further compromising the BBB and promoting neuroinflammation.^75^ This supports the hypothesis that gut microbiota dysbiosis is not merely a consequence of AD but an active contributor to its progression.

### Alterations in SCFA Profiles in 3 × Tg-AD Mice

**Figure 9:**
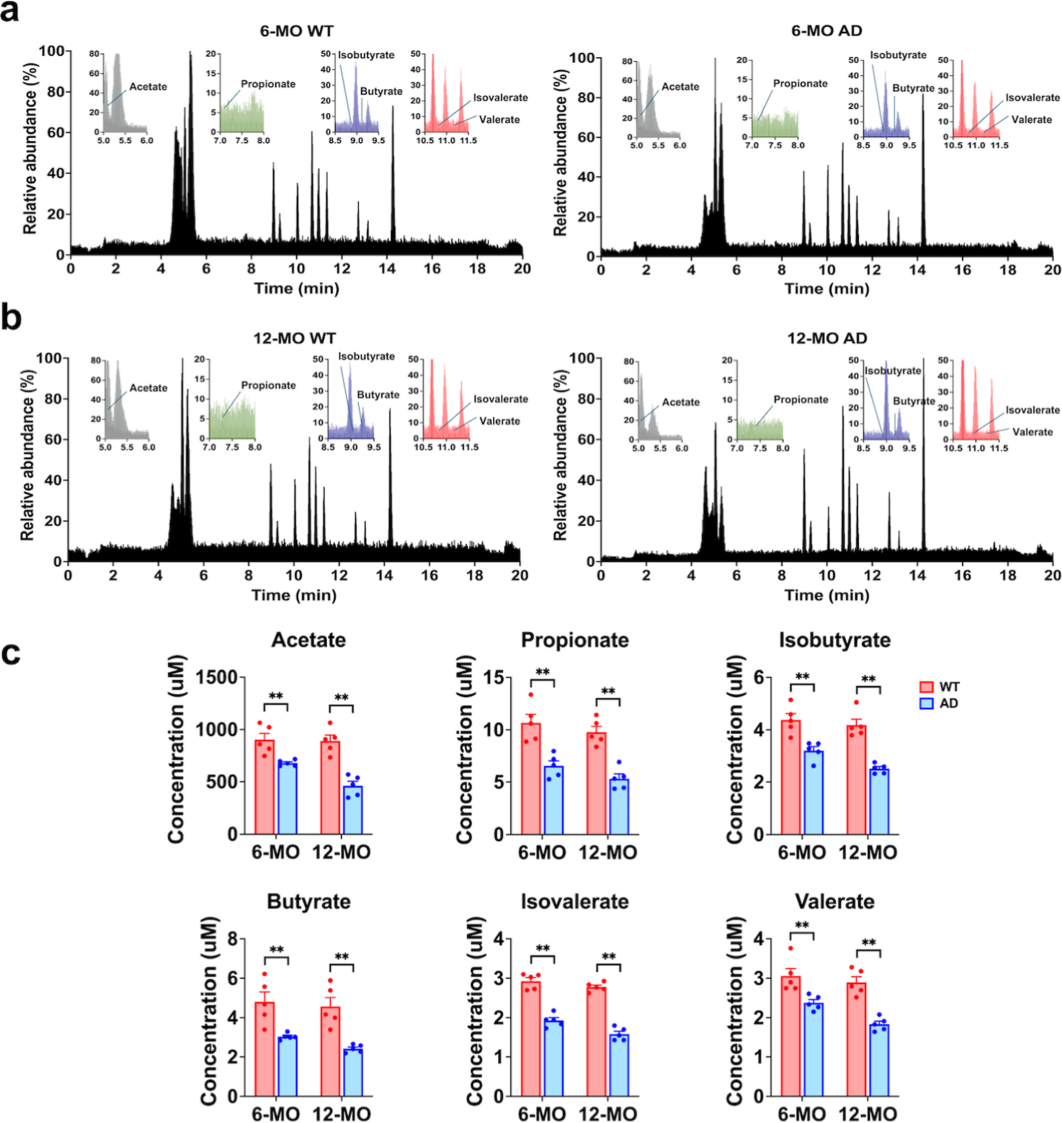
Analysis of serum SCFAs in WT and AD mice at 6 and 12-MO. (a) LC–MS/MS chromatograms of serum SCFAs from 6-MO WT and AD mice show relative abundance patterns of major SCFAs, including acetate, propionate, butyrate, isobutyrate isovalerate, and valerate. Insets illustrate retention times for specific SCFAs, highlighting reduced peak intensities in AD mice compared to WT controls. (b) LC–MS/MS chromatograms of serum SCFAs from 12-MO WT and AD mice reveal a similar trend, with marked reductions in SCFA levels in AD mice. Insets provide a magnified view of SCFA-specific retention times, demonstrating a notable decline in acetate, propionate, and butyrate abundance. (c) Quantitative analysis of SCFA concentrations, including acetate, propionate, isobutyrate, butyrate, isovalerate, and valerate, in WT and AD mice at 6-MO and 12-MO. Significant reductions in all six SCFAs were observed in AD mice at two time points compared to WT controls (**p < 0.01, FDR-adjusted Mann–Whitney *U* test). These findings suggest a progressive decline in serum SCFA levels during the early stages of AD pathology. Data are presented as mean ± SEM, with individual data points representing measurements from different mice.

SCFAs, microbial metabolites that play crucial roles in maintaining gut homeostasis, gut-brain communication, and systemic anti-inflammatory responses, were analyzed in 3 × Tg-AD and WT mice at 6 and 12-MO (Figure 9). SCFAs quantified included acetate, propionate, isobutyrate, butyrate, isovalerate, and valerate, providing insights into the metabolic consequences of gut microbiota dysbiosis associated with AD progression (see Table S1 in Supporting Information Note 2).

At 6-MO, SCFA profiles were comparable between the AD and WT groups (Figure 9a), with significant differences in the concentrations of acetate, propionate, isobutyrate, butyrate, isovalerate, and valerate (**p < 0.01, Mann–Whitney *U* test) (Figure 9c). This suggests that SCFA production and microbial metabolism remain largely affected in the early stages of AD. By 12-MO (Figure 9b), significant reductions were also observed in all six SCFAs concentrations in the AD group compared to WT controls (**p < 0.01, Mann–Whitney *U* test) (Figure 9c). These findings suggest a gradual decline in serum SCFA levels during the early stages of AD pathology, which continues into later stages of aging.

At 6-MO, significant reductions in all six SCFAs were already evident in the AD groups, indicating that microbial dysfunction and its impact on SCFA production commence early in the disease process. However, the minimal alterations in alpha and beta diversity and the stable *F*/*B* ratio were observed at this stage. These results suggest that early microbial shifts impair SCFA synthesis but do not yet lead to the severe metabolic deficits observed later. By 12-MO, gut dysbiosis becomes markedly pronounced, as evidenced by substantial and widespread declines in all six SCFAs, indicative of an advanced disruption in microbial fermentation pathways. *Firmicutes*, a major phylum associated with SCFA synthesis, particularly butyrate and branched-chain fatty acids,^83^ demonstrated decreased relative abundance in the 12-MO AD group, underscoring the profound shifts in microbial composition and functionality.

While acetate and butyrate are widely recognized for their roles in gut health,^84^ the observed reductions in propionate, isobutyrate, isovalerate, and valerate further reveal the widespread metabolic dysfunction associated with the progressive gut microbiota alterations. Acetate, the most abundant SCFA, serves as a precursor for butyrate production and supports epithelial integrity.^85^ Propionate modulates hepatic gluconeogenesis, reduces cholesterol levels, and exhibits potent immunomodulatory properties, while its depletion could exacerbate metabolic imbalances and inflammation.^86^ Butyrate, extensively studied for its anti-inflammatory, is a primary energy source for colonic epithelial cells, enhances tight junction integrity,^87^ and suppresses systemic inflammation via histone deacetylase (HDAC) inhibition, modulating gene expression linked to inflammatory and neuroprotective pathways.^15,88−91^ The reductions in isobutyrate and isovalerate, products of amino acid fermentation, suggest impaired protein metabolism and diminished microbial diversity,^92^ while valerate, though less studied, is implicated in epithelial repair, anti-inflammatory processes and neuronal activity.^93^ Collectively, the depletion of these SCFAs reflects a profound disruption of microbial metabolic pathways.

SCFAs are essential not only for gut health but also for brain function, influencing neurotransmitter synthesis and neuronal signaling.^16^ Butyrate, a major SCFA produced by gut bacteria such as *Clostridium*, *Ruminococcus*, *Eubacterium*, and *Faecalbacterium*, exerts neuroprotective effects through its HDAC inhibitor, enhancing neuronal plasticity, synaptic function, and neurogenesis.^94^ Studies have shown that butyrate’s HDAC inhibitory effects improve dopaminergic signaling, as evidenced by increased striatal dopamine levels in neurotoxicity models^95^ and protect dopaminergic neurons from damage in PD models, where it also reduces oxidative stress.^96^ Butyrate has also been found to attenuate neuronal apoptosis in ischemic stroke models.^97^ These neuroprotective effects are partly mediated through G protein-coupled receptors, including FA3R, a receptor expressed on enteroendocrine cells, including enteric neurons, which helps mitigate neurodegenerative damage, such as that caused by salsolinol-induced dopaminergic toxicity.^98^ Acetate is critical for synthesizing neurotransmitters like glutamate and GABA, maintaining the balance of excitatory and inhibitory signaling in the brain, which is vital for cognitive function.^99^ Propionate modulates dopaminergic pathways, which are key for regulating mood, motivation, and cognitive processes.^100^ Disruptions in SCFA levels, as seen in our study, may lead to imbalances in neurotransmitter systems, contributing to neurodegeneration and cognitive decline, as observed in AD. Strategies aimed at restoring SCFA levels, such as dietary interventions, probiotics, prebiotics, or direct SCFA supplementation, particularly targeting butyrate production, may mitigate gut dysbiosis, restore neuronal signaling, and slow neurodegenerative processes.^101,102^

### Role of Gut Microbiota Dysbiosis, SCFA Depletion in AD Progression

The findings from this study highlight the intricate interplay between gut microbiota dysbiosis, SCFA metabolism, and AD progression. By focusing on the significant reductions in all six SCFAs—acetate, propionate, isobutyrate, butyrate, isovalerate, and valerate—and the observed shifts in microbiota composition, this study adds a novel dimension to our understanding of AD pathology through gut-brain communication. The progression of gut microbiota dysbiosis in 3 × Tg-AD mice, particularly the reduction in the *F*/*B* ratio and significant beta diversity differences observed at 12-MO, aligns with the emergence of systemic inflammation and neuroinflammation. The global loss of SCFAs is of particular interest due to their established roles in gut health, systemic inflammation regulation, and neuroprotection.^15,88−90^ Acetate, the most abundant SCFA, plays a role in gut epithelial integrity and modulates regulatory T cells to suppress pro-inflammatory cytokine production, while propionate contributes to metabolic regulation and immune modulation, further emphasizing the significance of their depletion in driving inflammation.^88,103^

The absence of increased pro-inflammatory SCFAs in this study likely reflects the advanced dysbiosis characteristic of AD, where overall SCFA production is globally reduced. This reduction shifts the microbial metabolic balance toward inflammatory pathways such as LPS production and cytokine-mediated inflammation.^104^ Previous studies consistently report a reduction in beneficial SCFAs, including butyrate and acetate, correlating with disease progression and cognitive decline.^90,105^ While some research has suggested pro-inflammatory SCFAs may contribute to neuroinflammation, such pathways appear less relevant in this context of advanced dysbiosis, as protective SCFAs are already severely depleted.^16^ Instead, the findings suggest that the dominant mechanism involves the loss of anti-inflammatory SCFAs, which exacerbates systemic and neuroinflammation.^106^ Furthermore, gut barrier dysfunction and microbial metabolic shifts may suppress SCFA bioavailability, amplifying systemic inflammation and facilitating translocation of pro-inflammatory molecules into circulation.^16^

These findings emphasize the importance of restoring microbial homeostasis and SCFA production to mitigate inflammation and neurodegeneration in AD. Reduced levels of butyrate compromise tight junctions in the gut epithelium, increasing gut permeability and allowing microbial components such as LPS to enter the circulation, triggering systemic inflammation and further contributing to BBB dysfunction and neuroinflammation.^107,108^ This systemic inflammation, combined with the global depletion of SCFAs, leads to unchecked microglial activation and the release of pro-inflammatory cytokines such as IL-1β, IL-6, and TNFα, which amplify neuronal damage and synaptic dysfunction.^10,109^ While butyrate plays a central role as an HDAC inhibitor, its depletion reduces the brain’s capacity to counteract neurodegeneration.^110,111^ The other SCFAs—acetate, propionate, isobutyrate, isovalerate, and valerate—are equally essential for gut-brain homeostasis, and their collective depletion exacerbates disruptions in gut barrier function, immune signaling, and neurotransmitter metabolism, ultimately amplifying systemic inflammation, neuroinflammation, synaptic dysfunction, and the cognitive and behavioral deficits characteristic of AD.^84,99^

The results suggest that restoring microbial balance and SCFA production through dietary interventions, probiotics, or direct SCFA supplementation could mitigate systemic inflammation and preserve gut and brain homeostasis.^84^ The reduction in SCFAs at 12-MO indicates that interventions aimed at increasing SCFA levels could be most effective in the intermediate to advanced stages of AD, where dysbiosis and inflammation are most pronounced.^112^ These findings highlight SCFAs as both biomarkers of AD progression and potential therapeutic targets.^101,102^ Unlike previous studies focusing primarily on microbiota composition, this work provides mechanistic insights into how the depletion of SCFAs disrupts protective pathways, exacerbates systemic inflammation, and accelerates cognitive decline.

## Conclusions

This study establishes a robust connection between gut microbiota dysbiosis, systemic inflammation, and neuroinflammation in the progression of AD. Through behavioral assessments, DKI, gut microbiota profiling, SCFA quantification, and cytokine analysis, we elucidate the intricate interplay between the gut and brain in AD pathogenesis. AD mice exhibited significant memory deficits and microstructural brain alterations detected by DKI metrics, indicative of neuroinflammation and microstructural damage. Elevated systemic pro-inflammatory cytokines, including IL-1β, IL-6, TNFα, and IFN-γ, were correlated with increased microglial and astrocytic activity in memory-related brain regions, emphasizing the role of systemic inflammation in driving AD pathology. Gut microbiota analysis revealed significant beta diversity differences and a reduced *F*/*B* ratio in 12-MO AD mice, accompanied by a widespread depletion of SCFAs—including acetate, propionate, isobutyrate, butyrate, isovalerate, and valerate—reflecting profound disruptions in microbial metabolic function. This global reduction in SCFAs likely exacerbates gut barrier dysfunction, systemic inflammation, and neuroinflammation, reinforcing the bidirectional cycle of gut-brain axis dysfunction in AD. The integration of DKI as a biomarker for neuroinflammation and the identification of global SCFA depletion as a key metabolic deficit provide novel insights into AD pathogenesis. Targeting gut microbiota through dietary interventions, probiotics, or SCFAs supplementation offers a promising therapeutic strategy to mitigate systemic and neuroinflammation and slow AD progression. These findings highlight the critical role of gut-brain axis dysfunction in AD and pave the way for microbiota-targeted approaches to improve diagnosis, treatment, and disease management.

## Materials and Methods

### Animals

The current study employed male 3 × Tg-AD mice, a transgenic model widely recognized for its close genetic and pathological resemblance to human AD.^66,67^ These mice harbor mutations in APP, PS1, and MAPT genes, making them a robust model for investigating the progression of AD pathology. Two age groups, 6 and 12-MO old, were selected to represent early and midstages of disease progression, respectively, and were categorized into the AD group. Age-matched male B6129SF1/J mice, genetically unaltered and without predisposition to neurodegenerative disease, were utilized as the WT control group to provide baseline comparisons.

A total of 20 animals (*N* = 5 per group) were housed in the Laboratory Animal Center of Taipei Medical University under standardized conditions. The environment was maintained on a 12 h light–dark cycle, with a room temperature of 20 ± 3 °C and a relative humidity of 40–60%. Animals were provided ad libitum access to standard laboratory chow and filtered water to ensure optimal health and welfare during the study. The body weight of all mice at the time of experimentation was 20 ± 5 g, with no significant differences between groups.

All experimental procedures were conducted in compliance with the ethical standards and protocols approved by the Institutional Animal Care and Use Committee of Taipei Medical University (approval ID: LAC-2020-0286) in accordance with international guidelines for the care and use of laboratory animals. Additionally, regular health monitoring was conducted to ensure the welfare of the animals throughout the experimental timeline.

### Animal Grouping and Experimental Design

**Figure 10:**
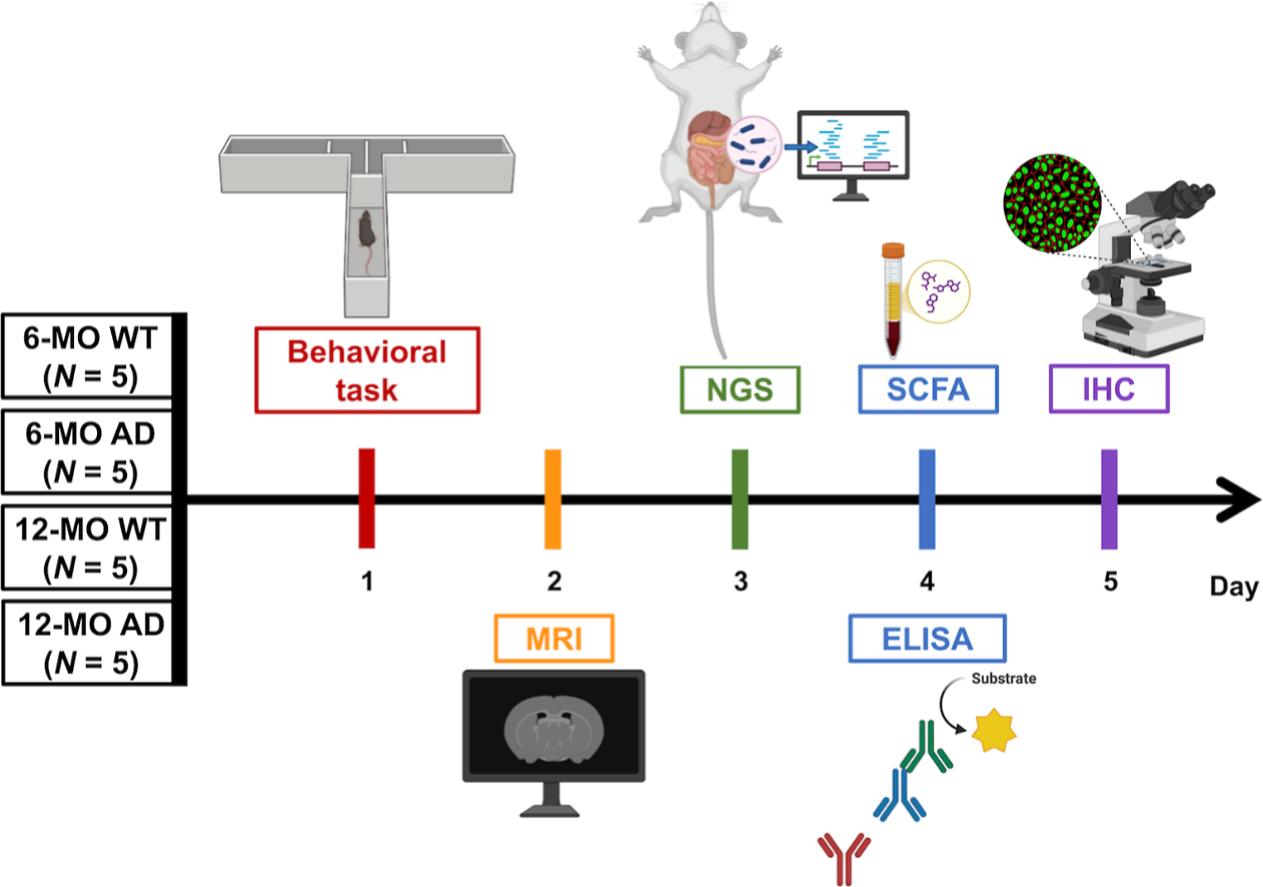
Comprehensive experimental design for assessing AD pathology in mice. The timeline illustrates the multistep workflow for analyzing behavioral, imaging, microbiota, cytokine, and neuroinflammatory changes in 6-MO and 12-MO WT and AD mice (*N* = 5 per group). On day 1, cognitive performance was evaluated using the T-maze task to assess working memory. On day 2, MRI/DKI was conducted to analyze microstructural brain changes. On day 3, fecal samples were processed with NGS to evaluate gut microbiota composition and diversity. On day 4, ELISA was used to quantify systemic pro-inflammatory cytokines, including IL-1β, IL-6, TNFα, and IFN-γ. Meanwhile, the serum samples were quantified for SCFA levels to assess dysregulated gut metabolism. On day 5, IHC was performed to assess microglial (Iba1) and astrocytic (GFAP) activation in key brain regions such as the mPFC, HIPP, and STR. This comprehensive workflow integrates multiple methodologies to investigate age- and disease-specific changes in AD pathology.

A total of 20 mice were randomly assigned to four experimental groups based on genotype and age: (1) 6-MO WT group, *N* = 5, (2) 6-MO AD group, *N* = 5, (3) 12-MO WT group, *N* = 5, and (4) 12-MO AD group, *N* = 5. Each group consisted of 5 animals. A schematic overview of the experimental design is illustrated in Figure 10.

The experimental protocol was standardized across all groups to ensure consistency. On day 1, the T-maze test was conducted to assess cognitive function, focusing on spatial learning and memory performance. On day 2, DKI scans were performed to evaluate microstructural changes in the brain. Stool samples were collected on day 3 to analyze gut microbiota composition and diversity. Blood samples were obtained on day 4 to measure circulating cytokine levels as markers of systemic inflammation as well as the analysis of SCFA. Finally, on day 5, animals were sacrificed under anesthesia for tissue collection, and IHC analysis was performed to investigate neuroinflammatory and pathological changes.

### Behavioral Test—T-Maze

Prior to the experiment, each mouse was acclimatized to the experimental room for 5–10 min to minimize stress. During the sample phase, the mouse was placed in the starting area with the Guillotine door closed for a duration of 10 s. The Guillotine door was then opened, allowing the mouse to explore and select one of the goal arms. Upon reaching a goal arm, the corresponding Guillotine door was partially closed, confining the mouse in the chosen arm for 30 s.

In the subsequent choice phase, the mouse was returned to the starting area without the Guillotine door to enable unrestricted movement. The mouse could freely select one of the goal arms. A different choice between the sample and choice phases indicated intact working memory, reflecting the animal’s capacity to alternate between goal arms in search of resources. Accuracy rate was calculated as the percentage of correct alternations between the two goal arms, with the baseline established based on choices made during the sample phase.

### DKI MRI Acquisition and Analysis

MRI data were acquired using a 7 T scanner equipped with a 30 cm diameter bore (Bruker Biospec 70/30 USR, Ettlingen, Germany). A planar surface coil (T7399 V3; Bruker Corp., Billerica, MA, USA) was positioned over the mouse’s head to receive the radio frequency (RF) signals, while RF pulses were transmitted via a linear volume coil. During scanning, mice were anesthetized using 3% isoflurane (Attane Isoflurane, Minrad Inc., NY, USA) in a mixture of 20% oxygen, 75% nitrogen, and 5% carbon dioxide. Each mouse was secured on a custom-designed animal holder, and respiratory rate was monitored using a pressure sensor (SA Instruments, Inc., New York, NY, USA) placed beneath the abdomen to ensure stable breathing at 45–50 breaths per min.

Field homogeneity was optimized using fast map shimming with first-order adjustments over an isotropic voxel (7 × 7 × 7 mm^3^). High-resolution anatomical images were acquired using T2-weighted rapid acquisition with relaxation enhancement (RARE) sequences [TR = 2500 ms, TE = 33 ms, matrix size = 256 × 256, field of view (FOV) = 20 × 20 mm^2^, number of excitations (NEX) = 4]. Diffusion-weighted images were obtained using a double-shot spin–echo planar imaging (EPI) protocol [TR = 3750 ms, TE = 31.2 ms, matrix size = 50 × 50, FOV = 20 × 20 mm^2^, slice thickness = 0.4 mm, 15 horizontal slices, and *b*-values = 0, 1,000, and 2000 s/mm^2^ across 30 diffusion directions and 10 nondiffusion directions].

DKI data were processed using custom MATLAB-based software (R2019a, The MathWorks Inc., Natick, MA, USA) to generate MK maps. Motion correction was performed using FSL (FMRIB Software Library, https://fsl.fmrib.ox.ac.uk/fsl/fslwiki), with coregistration of diffusion-weighted images. Region-based analysis was conducted in six ROIs associated with Papez circuit, including the mPFC, NAc, EC, Fornix, HIPP and STR. The identification of brain regions was guided by the C57Bl/6J mouse atlas and the Allen Mouse Brain Atlas. Diffusion metrics, including MK, AK, and RK, were calculated for each ROI.

The DKI processing workflow consisted of several key steps. Preprocessing began with motion and eddy current correction, implemented using FMRIB’s Linear Image Registration Tool (FLIRT). A rigid-body transformation was applied to correct for subject motion, while eddy current distortions were adjusted using a gradient reorientation process to maintain diffusion tensor accuracy. Framewise displacement (FD) thresholds were used to detect and remove excessive motion outliers, ensuring data integrity before further analysis.^113,114^

To account for susceptibility-induced distortions, B0 field mapping correction was applied, using nonlinear iterative field estimation to reduce EPI-related geometric distortions, especially in regions near the hippocampus and ventricular structures. The distortion correction was optimized to maintain alignment between diffusion-weighted and anatomical images, improving spatial accuracy in ROI-based analyses.^115,116^

For diffusion kurtosis model fitting, we employed a robust nonlinear least-squares estimation approach via the diffusion imaging in Python (DIPY) library. To mitigate partial volume effects (PVE) caused by cerebrospinal fluid (CSF) contamination, a two-compartment tensor model was applied, distinguishing free water from parenchymal diffusion and reducing bias in kurtosis estimates. This step was critical for regions adjacent to the ventricles and subcortical structures, where CSF infiltration could skew diffusion parameters.^117^

ROI selection and analysis were performed using a multistage registration approach. First, an atlas-based template was aligned to subject-specific diffusion space using nonlinear registration (ANTs) to define key brain regions. ROIs were drawn in native diffusion space, avoiding interpolation errors from resampling.^118,119^ Mean DKI values (MK, RK, AK) were extracted from All 6 predefined anatomical ROIs. ROI selection was guided by previous findings on neuroinflammation and microstructural alterations in AD, ensuring targeted analysis of memory and executive function-related areas.^120,121^

### Stool Sample Collection and Storage

Stool samples were collected immediately after DKI scanning for microbiota analysis at 6 and 12-MO of age. Samples were obtained directly without contact contamination using sterile gloves (Anderson Medicare, Changhua, Taiwan). Mice were gently restrained, and samples were collected into sterile microcentrifuge tubes (QIAGEN, Hilden, Germany). Collected samples were snap-frozen and stored at −80 °C for subsequent DNA extraction and microbiota profiling.

### DNA Extraction for Stool Samples

Stool samples were thawed and processed using the QIAamp DNA Stool Mini Kit (QIAGEN, Hilden, Germany) according to the manufacturer’s protocol. Samples were dissolved in lysis buffer, and genomic DNA was extracted with a final concentration of at least 5 ng/μL. DNA extracts were stored at −20 °C for downstream next-generation sequencing (NGS) applications.

### NGS Sequencing for Bacterial 16S rRNA

Microbial diversity was analyzed through 16S rRNA sequencing using universal primers targeting the V3–V4 hypervariable region (341F and 805R). Amplicon libraries were prepared using the Nextera XT Index Kit (Illumina Inc., San Diego, CA, USA), incorporating Illumina adapter and dual-index barcode sequences. Library quality and quantity were assessed using a QSep100 analyzer (BiOptic Inc., New Taipei City, Taiwan). Equimolar libraries were pooled and sequenced on an Illumina MiSeq platform with 300 bp paired end reads.

### Multiplex ELISA for Serum Cytokine Levels

Serum samples were obtained by centrifuging collected blood at 12,000 rpm for 20 min and stored at −80 °C. Cytokine levels (IL-1β, IL-6, IFN-γ, and TNFα) were quantified using a multiplex ELISA kit (MEK1016; Boster, Pleasanton, CA, USA). The assay employed a 96-well plate sandwich enzyme immunoassay technique, using analyte-specific antibodies and streptavidin-horseradish peroxidase (SHRP). Chemiluminescent substrates were added for quantification, with cytokine concentrations proportional to SHRP signal intensity.

### Quantification of Serum SCFAs

#### Derivatization Protocol for Serum Samples

To prepare serum samples for SCFA quantification, 50 μL of serum was mixed with 20 μL of a 200 mM solution of 3-nitrophenylhydrazine (3NPH) dissolved in 100% aqueous methanol and 20 μL of a 120 mM EDC solution containing 6% pyridine in 100% aqueous methanol. The mixture was incubated at 40 °C for 30 min to ensure the completion of the derivatization process. After derivatization, the reaction mixture was diluted with 10% aqueous methanol to a total volume of 210 μL. From this diluted sample, 75 μL was combined with 25 μL of an isotope-labeled internal standard (IS). A 10 μL aliquot of the prepared mixture was subsequently injected into the LC–MS/MS system for SCFA analysis.

#### Preparation of Isotope-Labeled Internal Standard Solution

An internal standard solution was prepared by dissolving a composite SCFA standard containing 20 mM acetic acid, 10 mM propionic acid, and 5 mM of seven other SCFAs in a microtube containing 1 mg of 13C6–3NPH _HCl. To initiate the labeling reaction, 20 μL of a 120 mM EDC solution with 6% pyridine in methanol was added. The mixture was incubated at 40 °C for 30 min, followed by dilution to a final volume of 100 mL using 10% aqueous methanol. This solution, when stored at −20 °C, remained stable for up to three months and was used for precise quantification of SCFAs. The product ions were measured in negative mode as [M – H]^−^. Details on their retention times on the LC column, as well as the collision energies and tube lens settings used for generating the product ions, can be found in Table S1 in Supporting Information Note 2.

#### Liquid Chromatography–Mass Spectrometry (LC–MS) Analysis

The SCFA quantification was performed using a Waters ACQUITY UPLC system (Waters Corporation, Milford, MA, USA) integrated with a Thermo Finnigan TSQ Quantum Ultra triple-quadrupole mass spectrometer. Data acquisition and control were managed via Xcalibur software. Chromatographic separation was achieved on an ACQUITY UPLC BEH C18 analytical column (130 Å, 1.7 μm, 2.1 × 100 mm), preceded by a precolumn filter (1.7 μm, 2.1 × 50 mm). The mobile phase consisted of solvent A (0.1% acetic acid in water) and solvent B (0.1% acetic acid in acetonitrile). A gradient flow was applied at 300 μL/min with the following program: starting with 10% solvent B (0.0–3.0 min), linearly increasing to 55% B by 17.0 min, transitioning to 100% B from 17.1 to 18.1 min, and re-equilibrating to 10% B between 18.5 and 20.0 min. The column temperature was held constant at 40 °C. The mass spectrometer was configured for electrospray ionization (ESI) in positive mode. Optimized operational parameters included a spray voltage of 3000 V, sheath gas (N_2_) pressure of 28 psi, auxiliary gas (N_2_) pressure of 10 psi, a capillary temperature of 350 °C, and a collision gas (Ar) pressure of 1.0 mTorr. These conditions ensured robust and reproducible detection of SCFAs with high sensitivity and accuracy.

### IHC for Neuroinflammation

Mice were perfused with 100 mL of normal saline followed by 4% paraformaldehyde in phosphate-buffered saline (PBS) for 10 min. Brains were postfixed in paraformaldehyde overnight and transferred to 30% sucrose for cryoprotection. Coronal sections (14 μm thick) were prepared and incubated in blocking buffer (0.2% Triton X-100 and 10% normal goat serum) for 1 h to prevent nonspecific binding. Primary antibodies targeting ionized calcium-binding adapter molecule 1 (Iba-1, 1:500; Wako, Richmond, VA, USA) and glial fibrillary acidic protein (GFAP, 1:500; Invitrogen, Camarillo, CA, USA) were applied overnight at 4 °C. Sections were then incubated with secondary antibodies conjugated with Alexa Fluor 568 and Alexa Fluor 488 (1:500; ThermoFisher, Waltham, MA, USA) and counterstained with DAPI.

### Statistical Analysis

Data were expressed as mean ± standard error of the mean (SEM). Behavioral and DKI data were analyzed using the Mann–Whitney *U* test (*p* < 0.05). For IHC analysis, normalized counts of Iba-1+ and GFAP + cells were compared using the Mann–Whitney *U* test. Beta diversity of microbiota composition was assessed using the *adonis* and *betadisper* functions in the vegan package (v2.4) of R. To mitigate the potential inflation of Type I errors from multiple comparisons, the false discovery rate (FDR) correction was used to adjust significance levels following the Mann–Whitney *U* test. The corresponding effect size and power values were determined using the open-source tool-box, G*Power (Version 3.1.9.2, Institut für Experimentelle Psychologie, Dusseldorf, Germany),^122−124^ which are shown in Tables S2–S8 in Supporting Information Note 3 for all variables measured in the study. All statistical analyses were performed using GraphPad Prism version 9, and were verified using SPSS version 20.0 (SPSS Inc., Chicago, IL, USA). The results are reported as the mean ± standard error of the mean (SEM).

## Supporting Information Available

Additional experimental details, imaging analyses, and statistical measures: FS1: white matter connectivity analysis; TS1: SCFA quantification; TS2: behavioral effect sizes and power; TS3: DKI effect sizes and power; TS4: fiber-tract effect sizes and power; TS5: IHC effect sizes and power; TS6: cytokine effect sizes and power; TS7: gut microbiota effect sizes and power; TS8: SCFA effect sizes and power (PDF)

## Notes

The experiment procedures were approved by the Institutional Animal Care and Use Committee of the Taipei Medical University (approval ID/number: LAC-2020-0286). Some of the graphical parts of the figures were created using Biorender.com.

## References

[ref1] Kumar A.; Alzheimer Disease. In StatPearls; StatPearls Publishing LLC.: Treasure Island (FL), 2025.

[ref2] Tcw J.; Goate A. M. Genetics of β-Amyloid Precursor Protein in Alzheimer’s Disease. Cold Spring Harbor Perspect. Med. 2017, 7 (6), a024539 10.1101/cshperspect.a024539. PMC5453386 28003277

[ref3] Cai Y.; et al. Microglia in the Neuroinflammatory Pathogenesis of Alzheimer’s Disease and Related Therapeutic Targets. Front. Immunol. 2022, 13, 856376 10.3389/fimmu.2022.856376. 35558075 PMC9086828

[ref4] Lian H.; et al. NFκB-activated astroglial release of complement C3 compromises neuronal morphology and function associated with Alzheimer’s disease. Neuron 2015, 85 (1), 101–115. 10.1016/j.neuron.2014.11.018. 25533482 PMC4289109

[ref5] Wei Y.; et al. The complement C3-C3aR pathway mediates microglia-astrocyte interaction following status epilepticus. Glia 2021, 69 (5), 1155–1169. 10.1002/glia.23955. 33314324 PMC7936954

[ref6] Kamagata K.; et al. Advanced diffusion magnetic resonance imaging in patients with Alzheimer’s and Parkinson’s diseases. Neural Regener. Res. 2020, 15 (9), 1590–1600. 10.4103/1673-5374.276326. PMC7437577 32209758

[ref7] Gullotta G. S.; Costantino G.; Sortino M. A.; Spampinato S. F. Microglia and the Blood-Brain Barrier: An External Player in Acute and Chronic Neuroinflammatory Conditions. Int. J. Mol. Sci. 2023, 24 (11), 9144 10.3390/ijms24119144. 37298096 PMC10252410

[ref8] Jensen J. H.; et al. Diffusional kurtosis imaging: the quantification of non-gaussian water diffusion by means of magnetic resonance imaging. Magn. Reson. Med. 2005, 53 (6), 1432 10.1002/mrm.20508. 15906300

[ref9] Kamatham P. T.; et al. Pathogenesis, diagnostics, and therapeutics for Alzheimer’s disease: Breaking the memory barrier. Ageing Res. Rev. 2024, 101, 102481 10.1016/j.arr.2024.102481. 39236855

[ref10] Solano M. V. M.; Lara C. S.; Sánchez-Garibay C.; Soto-Rojas L. O.; Escobedo-Ávila I.; Tena-Suck M. L.; Ortíz-Butrón R.; Choreño-Parra J. A.; Romero-López J. P.; Camargo M. E. M. Effect of Systemic Inflammation in the CNS: A Silent History of Neuronal Damage. Int. J. Mol. Sci. 2023, 24 (15), 11902 10.3390/ijms241511902. 37569277 PMC10419139

[ref11] Kozhakhmetov S.; Kaiyrlykyzy A.; Jarmukhanov Z.; Vinogradova E.; Zholdasbekova G.; Alzhanova D.; Kunz J.; Kushugulova A.; Askarova S. Inflammatory Manifestations Associated With Gut Dysbiosis in Alzheimer’s Disease. Int. J. Alzheimers Dis. 2024, 2024, 9741811 10.1155/2024/9741811. 39346576 PMC11436273

[ref12] Loh J. S.; Mak W. Q.; Tan L. K. S.; Ng C. X.; Chan H. H.; Yeow S. H.; Foo J. B.; Ong Y. S.; How C. W.; Khaw K. Y. Microbiota–gut–brain axis and its therapeutic applications in neurodegenerative diseases. Signal Transduct. Targeted Ther. 2024, 9 (1), 37 10.1038/s41392-024-01743-1. PMC10869798 38360862

[ref13] Shukla P. K.; et al. Alterations in the Gut-Microbial-Inflammasome-Brain Axis in a Mouse Model of Alzheimer’s Disease. Cells 2021, 10, 779 10.3390/cells10040779. 33916001 PMC8067249

[ref14] Di Vincenzo F.; et al. Gut microbiota, intestinal permeability, and systemic inflammation: a narrative review. Internal Emerg. Med. 2024, 19 (2), 275–293. 10.1007/s11739-023-03374-w. 37505311 PMC10954893

[ref15] Fusco W.; Lorenzo M. B.; Cintoni M.; Porcari S.; Rinninella E.; Kaitsas F.; Lener E.; Mele M. C.; Gasbarrini A.; Collado M. C.; et al. Short-Chain Fatty-Acid-Producing Bacteria: Key Components of the Human Gut Microbiota. Nutrients 2023, 15 (9), 2211 10.3390/nu15092211. 37432351 PMC10180739

[ref16] O’Riordan K. J.; et al. Short chain fatty acids: Microbial metabolites for gut-brain axis signalling. Mol. Cell. Endocrinol. 2022, 546, 111572 10.1016/j.mce.2022.111572. 35066114

[ref17] Kinney J. W.; et al. Inflammation as a central mechanism in Alzheimer’s disease. Alzheimer’s Dementia 2018, 4, 575–590. 10.1016/j.trci.2018.06.014. PMC6214864 30406177

[ref18] Caldito N. G. Role of tumor necrosis factor-alpha in the central nervous system: a focus on autoimmune disorders. Front. Immunol. 2023, 14, 1213448 10.3389/fimmu.2023.1213448. 37483590 PMC10360935

[ref19] Kim C. H. Complex regulatory effects of gut microbial short-chain fatty acids on immune tolerance and autoimmunity. Cell. Mol. Immunol. 2023, 20 (4), 341–350. 10.1038/s41423-023-00987-1. 36854801 PMC10066346

[ref20] Steven A. J.; Zhuo J.; Melhem E. R. Diffusion Kurtosis Imaging: An Emerging Technique for Evaluating the Microstructural Environment of the Brain. Am. J. Roentgenol. 2014, 202 (1), W26–W33. 10.2214/AJR.13.11365. 24370162

[ref21] Guglielmetti C.; et al. Diffusion kurtosis imaging probes cortical alterations and white matter pathology following cuprizone induced demyelination and spontaneous remyelination. Neuroimage 2016, 125, 363–377. 10.1016/j.neuroimage.2015.10.052. 26525654 PMC4935929

[ref22] Falangola M. F.; Nie X.; Ward R.; McKinnon E. T.; Dhiman S.; Nietert P. J.; Helpern J. A.; Jensen J. H. Diffusion MRI detects early brain microstructure abnormalities in 2-month-old 3 × Tg-AD mice. NMR Biomed. 2020, 33 (9), e4346 10.1002/nbm.4346. 32557874 PMC7683375

[ref23] Struyfs H.; et al. Diffusion Kurtosis Imaging: A Possible MRI Biomarker for AD Diagnosis?. J. Alzheimers Dis. 2015, 48 (4), 937–948. 10.3233/JAD-150253. 26444762 PMC4927852

[ref24] Aske D. The correlation between Mini-Mental State Examination scores and Katz ADL status among dementia patients. Rehabil. Nurs. 1990, 15 (3), 140–142. 10.1002/j.2048-7940.1990.tb01456.x. 2343176

[ref25] Praet J.; Manyakov N. V.; Muchene L.; Mai Z.; Terzopoulos V.; de Backer S.; Torremans A.; Guns P. J.; Van De Casteele T.; Bottelbergs A.; et al. Diffusion kurtosis imaging allows the early detection and longitudinal follow-up of amyloid-β-induced pathology. Alzheimers Res. Ther. 2018, 10 (1), 1 10.1186/s13195-017-0329-8. 29370870 PMC6389136

[ref26] Deacon R. M. J.; Rawlins J. N. P. T-maze alternation in the rodent. Nat. Protoc. 2006, 1 (1), 7–12. 10.1038/nprot.2006.2. 17406205

[ref27] Davis K. E.; Burnett K.; Gigg J. Water and T-maze protocols are equally efficient methods to assess spatial memory in 3xTg Alzheimer’s disease mice. Behav. Brain Res. 2017, 331, 54–66. 10.1016/j.bbr.2017.05.005. 28511979

[ref28] Arendash G. W.; et al. Multi-metric behavioral comparison of APPsw and P301L models for Alzheimer’s disease: linkage of poorer cognitive performance to tau pathology in forebrain. Brain Res. 2004, 1012 (1–2), 29–41. 10.1016/j.brainres.2004.02.081. 15158158

[ref29] Scheltens P.; et al. Alzheimer’s disease. Lancet 2021, 397 (10284), 1577–1590. 10.1016/S0140-6736(20)32205-4. 33667416 PMC8354300

[ref30] Wirt R. A.; Hyman J. M. Integrating Spatial Working Memory and Remote Memory: Interactions between the Medial Prefrontal Cortex and Hippocampus. Brain Sci. 2017, 7 (4), 43 10.3390/brainsci7040043. 28420200 PMC5406700

[ref31] Cordella A.; et al. Dopamine loss alters the hippocampus-nucleus accumbens synaptic transmission in the Tg2576 mouse model of Alzheimer’s disease. Neurobiol. Dis. 2018, 116, 142–154. 10.1016/j.nbd.2018.05.006. 29778899

[ref32] Al-Ezzi A.; Arechavala R. J.; Butler R.; Nolty A.; Kang J. J.; Shimojo S.; Wu D. A.; Fonteh A. N.; Kleinman M. T.; Kloner R. A.; et al. Disrupted brain functional connectivity as early signature in cognitively healthy individuals with pathological CSF amyloid/tau. Commun. Biol. 2024, 7 (1), 1037 10.1038/s42003-024-06673-w. 39179782 PMC11344156

[ref33] Adamu A.; Li S.; Gao F.; Xue G. The role of neuroinflammation in neurodegenerative diseases: current understanding and future therapeutic targets. Front. Aging Neurosci. 2024, 16, 1347987 10.3389/fnagi.2024.1347987. 38681666 PMC11045904

[ref34] Vanhoutte G.; et al. Diffusion kurtosis imaging to detect amyloidosis in an APP/PS1 mouse model for Alzheimer’s disease. Magn. Reson. Med. 2013, 69 (4), 1115–1121. 10.1002/mrm.24680. 23494926

[ref35] Ni R. Magnetic Resonance Imaging in Animal Models of Alzheimer’s Disease Amyloidosis. Int. J. Mol. Sci. 2021, 22 (23), 12768 10.3390/ijms222312768. 34884573 PMC8657987

[ref36] Chu X.; et al. Comparison of brain microstructure alterations on diffusion kurtosis imaging among Alzheimer’s disease, mild cognitive impairment, and cognitively normal individuals. Front. Aging Neurosci. 2022, 14, 919143 10.3389/fnagi.2022.919143. 36034135 PMC9416000

[ref37] Guglielmetti C.; et al. Diffusion kurtosis imaging probes cortical alterations and white matter pathology following cuprizone induced demyelination and spontaneous remyelination. Neuroimage 2016, 125, 363–377. 10.1016/j.neuroimage.2015.10.052. 26525654 PMC4935929

[ref38] Nelson M. R.; Keeling E. G.; Stokes A. M.; Bergamino M. Exploring white matter microstructural alterations in mild cognitive impairment: a multimodal diffusion MRI investigation utilizing diffusion kurtosis and free-water imaging. Front. Neurosci. 2024, 18, 1440653 10.3389/fnins.2024.1440653. 39170682 PMC11335656

[ref39] Dash U. C.; et al. Oxidative stress and inflammation in the pathogenesis of neurological disorders: Mechanisms and implications. Acta Pharm. Sin. B 2025, 15 (1), 15–34. 10.1016/j.apsb.2024.10.004. 40041912 PMC11873663

[ref40] Zhang H.; et al. Altered Microstructure of Cerebral Gray Matter in Neuromyelitis Optica Spectrum Disorder-Optic Neuritis: A DKI Study. Front. Neurosci. 2021, 15, 738913 10.3389/fnins.2021.738913. 34987355 PMC8720872

[ref41] Lee D. C.; et al. Review: experimental manipulations of microglia in mouse models of Alzheimer’s pathology: activation reduces amyloid but hastens tau pathology. Neuropathol. Appl. Neurobiol. 2013, 39 (1), 69–85. 10.1111/nan.12002. 23171029 PMC4300851

[ref42] Fruhwürth S.; Zetterberg H.; Paludan S. R. Microglia and amyloid plaque formation in Alzheimer’s disease – Evidence, possible mechanisms, and future challenges. J. Neuroimmunol. 2024, 390, 578342 10.1016/j.jneuroim.2024.578342. 38640827

[ref43] Escobar I.; Xu J.; Jackson C. W.; Perez-Pinzon M. A. Altered Neural Networks in the Papez Circuit: Implications for Cognitive Dysfunction after Cerebral Ischemia. J. Alzheim. Dis. 2018, 67, 425–446. 10.3233/jad-180875. PMC6398564 30584147

[ref44] Depp C.; et al. Myelin dysfunction drives amyloid-β deposition in models of Alzheimer’s disease. Nature 2023, 618 (7964), 349–357. 10.1038/s41586-023-06120-6. 37258678 PMC10247380

[ref45] Chen C.; Shu Y.; Yan C.; Li H.; Huang Z.; Shen S.; Liu C.; Jiang Y.; Huang S.; Wang Z.; et al. Astrocyte-derived clusterin disrupts glial physiology to obstruct remyelination in mouse models of demyelinating diseases. Nat. Commun. 2024, 15 (1), 7791 10.1038/s41467-024-52142-7. 39242637 PMC11379856

[ref46] Kim J.; et al. Pathological phenotypes of astrocytes in Alzheimer’s disease. Exp. Mol. Med. 2024, 56 (1), 95–99. 10.1038/s12276-023-01148-0. 38172603 PMC10834520

[ref47] Roth A. D.; Ramírez G.; Alarcón R.; Von Bernhardi R. Oligodendrocytes damage in Alzheimer’s disease: beta amyloid toxicity and inflammation. Biol. Res. 2005, 38 (4), 381–387. 10.4067/s0716-97602005000400011. 16579521

[ref48] De Strooper B.; Karran E. The Cellular Phase of Alzheimer’s Disease. Cell 2016, 164 (4), 603–615. 10.1016/j.cell.2015.12.056. 26871627

[ref49] Corbin-Stein N. J.; Childers G. M.; Webster J. M.; Zane A.; Yang Y. T.; Mudium N.; Gupta R.; Manfredsson F. P.; Kordower J. H.; Harms A. S. IFNγ drives neuroinflammation, demyelination, and neurodegeneration in a mouse model of multiple system atrophy. Acta Neuropathol. Commun. 2024, 12 (1), 11 10.1186/s40478-023-01710-x. 38238869 PMC10797897

[ref50] Yamamoto M.; et al. Interferon-gamma and tumor necrosis factor-alpha regulate amyloid-beta plaque deposition and beta-secretase expression in Swedish mutant APP transgenic mice. Am. J. Pathol. 2007, 170 (2), 680–692. 10.2353/ajpath.2007.060378. 17255335 PMC1851864

[ref51] Turner M. D.; et al. Cytokines and chemokines: At the crossroads of cell signalling and inflammatory disease. Biochim. Biophys. Acta Mol. Cell Res. 2014, 1843 (11), 2563–2582. 10.1016/j.bbamcr.2014.05.014. 24892271

[ref52] Zhang W.; Xiao D.; Mao Q.; Xia H. Role of neuroinflammation in neurodegeneration development. Signal Transduct. Targeted Ther. 2023, 8 (1), 267 10.1038/s41392-023-01486-5. PMC10336149 37433768

[ref53] Mendiola A. S.; Cardona A. E. The IL-1β phenomena in neuroinflammatory diseases. J. Neural Transm. 2018, 125 (5), 781–795. 10.1007/s00702-017-1732-9. 28534174 PMC5699978

[ref54] Wang W. Y.; et al. Role of pro-inflammatory cytokines released from microglia in Alzheimer’s disease. Ann. Transl. Med. 2015, 3 (10), 136 10.3978/j.issn.2305-5839.2015.03.49. 26207229 PMC4486922

[ref55] Rani V.; et al. Role of pro-inflammatory cytokines in Alzheimer’s disease and neuroprotective effects of pegylated self-assembled nanoscaffolds. Curr. Res. Pharmacol. Drug Discov. 2023, 4, 100149 10.1016/j.crphar.2022.100149. 36593925 PMC9804106

[ref56] Dugan L. L.; et al. IL-6 mediated degeneration of forebrain GABAergic interneurons and cognitive impairment in aged mice through activation of neuronal NADPH oxidase. PLoS One 2009, 4 (5), e5518 10.1371/journal.pone.0005518. 19436757 PMC2678193

[ref57] Hou L.; et al. Inhibition of NADPH oxidase by apocynin prevents learning and memory deficits in a mouse Parkinson’s disease model. Redox Biol. 2019, 22, 101134 10.1016/j.redox.2019.101134. 30798073 PMC6389731

[ref58] Whiten D. R.; Brownjohn P. W.; Moore S.; De S.; Strano A.; Zuo Y.; Haneklaus M.; Klenerman D.; Livesey F. J. Tumour necrosis factor induces increased production of extracellular amyloid-β- and α-synuclein-containing aggregates by human Alzheimer’s disease neurons. Brain Commun. 2020, 2 (2), fcaa146 10.1093/braincomms/fcaa146. 33543132 PMC7850285

[ref59] Plantone D.; Pardini M.; Righi D.; Manco C.; Colombo B. M.; De Stefano N. The Role of TNF-α in Alzheimer’s Disease: A Narrative Review. Cells 2023, 13 (1), 54 10.3390/cells13010054. 38201258 PMC10778385

[ref60] Zenaro E.; Piacentino G.; Constantin G. The blood-brain barrier in Alzheimer’s disease. Neurobiol. Dis. 2017, 107, 41–56. 10.1016/j.nbd.2016.07.007. 27425887 PMC5600438

[ref61] Zhang Y.; Chen H.; Li R.; Sterling K.; Song W. Amyloid β-based therapy for Alzheimer’s disease: challenges, successes and future. Signal Transduct. Targeted Ther. 2023, 8 (1), 248 10.1038/s41392-023-01484-7. PMC10310781 37386015

[ref62] McKenna F.; Miles L.; Donaldson J.; Castellanos F. X.; Lazar M. Diffusion kurtosis imaging of gray matter in young adults with autism spectrum disorder. Sci. Rep. 2020, 10 (1), 21465 10.1038/s41598-020-78486-w. 33293640 PMC7722927

[ref63] Dong J. W.; et al. Diffusion MRI biomarkers of white matter microstructure vary nonmonotonically with increasing cerebral amyloid deposition. Neurobiol. Aging 2020, 89, 118–128. 10.1016/j.neurobiolaging.2020.01.009. 32111392 PMC7314576

[ref64] Jensen J. H. Impact of intra-axonal kurtosis on fiber orientation density functions estimated with fiber ball imaging. Magn. Reson. Med. 2022, 88 (3), 1347–1354. 10.1002/mrm.29270. 35436362 PMC9246967

[ref65] Yang S.; Magnutzki A.; Alami N. O.; Lattke M.; Hein T. M.; Scheller J. S.; Kröger C.; Oswald F.; Yilmazer-Hanke D.; Wirth T.; et al. IKK2/NF-κB Activation in Astrocytes Reduces amyloid β Deposition: A Process Associated with Specific Microglia Polarization. Cells 2021, 10 (10), 2669 10.3390/cells10102669. 34685649 PMC8534251

[ref66] Shao F.; et al. Microglia and Neuroinflammation: Crucial Pathological Mechanisms in Traumatic Brain Injury-Induced Neurodegeneration. Front. Aging Neurosci. 2022, 14, 825086 10.3389/fnagi.2022.825086. 35401152 PMC8990307

[ref67] Wang C.; Zong S.; Cui X.; Wang X.; Wu S.; Wang L.; Liu Y.; Lu Z. The effects of microglia-associated neuroinflammation on Alzheimer’s disease. Front. Immunol. 2023, 14, 1117172 10.3389/fimmu.2023.1117172. 36911732 PMC9992739

[ref68] Zhu J.; et al. Performances of diffusion kurtosis imaging and diffusion tensor imaging in detecting white matter abnormality in schizophrenia. Neuroimage 2015, 7, 170–176. 10.1016/j.nicl.2014.12.008. 25610778 PMC4300008

[ref69] Szögi T.; et al. Examination of Longitudinal Alterations in Alzheimer’s Disease-Related Neurogenesis in an APP/PS1 Transgenic Mouse Model, and the Effects of P33, a Putative Neuroprotective Agent Thereon. Int. J. Mol. Sci. 2022, 23, 10364 10.3390/ijms231810364. 36142277 PMC9499399

[ref70] Yu B.; Shan Y.; Ding J. A literature review of MRI techniques used to detect amyloid-beta plaques in Alzheimer’s disease patients. Ann. Palliat. Med. 2021, 10 (9), 10062–10074. 10.21037/apm-21-825. 34551578

[ref71] Song J.; Cho E.; Lee H.; Lee S.; Kim S.; Kim J. Development of Neurodegenerative Disease Diagnosis and Monitoring from Traditional to Digital Biomarkers. Biosensors 2025, 15 (2), 102 10.3390/bios15020102. 39997004 PMC11852611

[ref72] Sochocka M.; et al. The Gut Microbiome Alterations and Inflammation-Driven Pathogenesis of Alzheimer’s Disease-a Critical Review. Mol. Neurobiol. 2019, 56 (3), 1841–1851. 10.1007/s12035-018-1188-4. 29936690 PMC6394610

[ref73] Wu S. C.; Cao Z. S.; Chang K. M.; Juang J. L. Intestinal microbial dysbiosis aggravates the progression of Alzheimer’s disease in Drosophila. Nat. Commun. 2017, 8 (1), 24 10.1038/s41467-017-00040-6. 28634323 PMC5478647

[ref74] Pan Q.; et al. Gut Microbiota Dysbiosis in Systemic Lupus Erythematosus: Novel Insights into Mechanisms and Promising Therapeutic Strategies. Front. Immunol. 2021, 12, 799788 10.3389/fimmu.2021.799788. 34925385 PMC8677698

[ref75] Zhao M. . a.; et al. Immunological mechanisms of inflammatory diseases caused by gut microbiota dysbiosis: A review. Biomed. Pharmacother. 2023, 164, 114985 10.1016/j.biopha.2023.114985. 37311282

[ref76] Yang F.; et al. Gut microbiota-derived short-chain fatty acids and hypertension: Mechanism and treatment. Biomed. Pharmacother. 2020, 130, 110503 10.1016/j.biopha.2020.110503. 34321175

[ref77] Ashique S.; et al. Gut-brain axis: A cutting-edge approach to target neurological disorders and potential synbiotic application. Heliyon 2024, 10 (13), e34092 10.1016/j.heliyon.2024.e34092. 39071627 PMC11279763

[ref78] Chakaroun R. M.; Massier L.; Kovacs P. Gut Microbiome, Intestinal Permeability, and Tissue Bacteria in Metabolic Disease: Perpetrators or Bystanders?. Nutrients 2020, 12 (4), 1082 10.3390/nu12041082. 32295104 PMC7230435

[ref79] Vogt N. M.; Kerby R. L.; Dill-McFarland K. A.; Harding S. J.; Merluzzi A. P.; Johnson S. C.; Carlsson C. M.; Asthana S.; Zetterberg H.; Blennow K.; et al. Gut microbiome alterations in Alzheimer’s disease. Sci. Rep. 2017, 7 (1), 13537 10.1038/s41598-017-13601-y. 29051531 PMC5648830

[ref80] Nogal A.; Valdes A. M.; Menni C. The role of short-chain fatty acids in the interplay between gut microbiota and diet in cardio-metabolic health. Gut Microbes 2021, 13 (1), 1–24. 10.1080/19490976.2021.1897212. PMC8007165 33764858

[ref81] Lukiw W. J. Bacteroides fragilis Lipopolysaccharide and Inflammatory Signaling in Alzheimer’s Disease. Front. Microbiol. 2016, 7, 1544 10.3389/fmicb.2016.01544. 27725817 PMC5035737

[ref82] Biddle A.; et al. Untangling the genetic basis of fibrolytic specialization by Lachnospiraceae and Ruminococcaceae in diverse gut communities. Diversity 2013, 5 (3), 627–640. 10.3390/d5030627.

[ref83] Recharla N.; Geesala R.; Shi X.-Z. Gut Microbial Metabolite Butyrate and Its Therapeutic Role in Inflammatory Bowel Disease: A Literature Review. Nutrients 2023, 15, 2275 10.3390/nu15102275. 37242159 PMC10221771

[ref84] Silva Y. P.; Bernardi A.; Frozza R. L. The Role of Short-Chain Fatty Acids From Gut Microbiota in Gut-Brain Communication. Front. Endocrinol. 2020, 11, 25 10.3389/fendo.2020.00025. PMC7005631 32082260

[ref85] Deleu S.; et al. Short chain fatty acids and its producing organisms: An overlooked therapy for IBD?. EBioMedicine 2021, 66, 103293 10.1016/j.ebiom.2021.103293. 33813134 PMC8047503

[ref86] Haghikia A.; et al. Propionate attenuates atherosclerosis by immune-dependent regulation of intestinal cholesterol metabolism. Eur. Heart J. 2022, 43 (6), 518–533. 10.1093/eurheartj/ehab644. 34597388 PMC9097250

[ref87] Salvi P. S.; Cowles R. A. Butyrate and the Intestinal Epithelium: Modulation of Proliferation and Inflammation in Homeostasis and Disease. Cells 2021, 10 (7), 1775 10.3390/cells10071775. 34359944 PMC8304699

[ref88] Venegas D. P.; De la Fuente M. K.; Landskron G.; González M. J.; Quera R.; Dijkstra G.; Harmsen H. J. M.; Faber K. N.; Hermoso M. A. Short Chain Fatty Acids (SCFAs)-Mediated Gut Epithelial and Immune Regulation and Its Relevance for Inflammatory Bowel Diseases. Front. Immunol. 2019, 10, 277 10.3389/fimmu.2019.00277. 30915065 PMC6421268

[ref89] Wen Y.; Yang L.; Wang Z.; Liu X.; Gao M.; Zhang Y.; Wang J.; He P. Blocked conversion of Lactobacillus johnsonii derived acetate to butyrate mediates copper-induced epithelial barrier damage in a pig model. Microbiome 2023, 11 (1), 218 10.1186/s40168-023-01655-2. 37777765 PMC10542248

[ref90] Korsten S.; Vromans H.; Garssen J.; Willemsen L. E. M. Butyrate Protects Barrier Integrity and Suppresses Immune Activation in a Caco-2/PBMC Co-Culture Model While HDAC Inhibition Mimics Butyrate in Restoring Cytokine-Induced Barrier Disruption. Nutrients 2023, 15 (12), 2760 10.3390/nu15122760. 37375664 PMC10305054

[ref91] O’Riordan K. J.; et al. Short chain fatty acids: Microbial metabolites for gut-brain axis signalling. Mol. Cell. Endocrinol. 2022, 546, 111572 10.1016/j.mce.2022.111572. 35066114

[ref92] Neis E. P.; Dejong C. H.; Rensen S. S. The role of microbial amino acid metabolism in host metabolism. Nutrients 2015, 7 (4), 2930–2946. 10.3390/nu7042930. 25894657 PMC4425181

[ref93] Yazici D.; et al. The epithelial barrier: The gateway to allergic, autoimmune, and metabolic diseases and chronic neuropsychiatric conditions. Semin. Immunol. 2023, 70, 101846 10.1016/j.smim.2023.101846. 37801907

[ref94] Zhang L.-Y.; et al. Role of histone deacetylases and their inhibitors in neurological diseases. Pharmacol. Res. 2024, 208, 107410 10.1016/j.phrs.2024.107410. 39276955

[ref95] Sharma S.; Taliyan R.; Singh S. Beneficial effects of sodium butyrate in 6-OHDA induced neurotoxicity and behavioral abnormalities: Modulation of histone deacetylase activity. Behav. Brain Res. 2015, 291, 306–314. 10.1016/j.bbr.2015.05.052. 26048426

[ref96] Paiva I.; et al. Sodium butyrate rescues dopaminergic cells from alpha-synuclein-induced transcriptional deregulation and DNA damage. Hum. Mol. Genet. 2017, 26 (12), 2231–2246. 10.1093/hmg/ddx114. 28369321

[ref97] Zhou Z.; et al. Sodium butyrate attenuated neuronal apoptosis via GPR41/Gβγ/PI3K/Akt pathway after MCAO in rats. J. Cereb. Blood Flow Metab. 2021, 41 (2), 267–281. 10.1177/0271678X20910533. 32151222 PMC8370004

[ref98] Getachew B.; et al. Butyrate Protects Against Salsolinol-Induced Toxicity in SH-SY5Y Cells: Implication for Parkinson’s Disease. Neurotox. Res. 2020, 38 (3), 596–602. 10.1007/s12640-020-00238-5. 32572814 PMC7484007

[ref99] Chen Y.; Xu J.; Chen Y. Regulation of Neurotransmitters by the Gut Microbiota and Effects on Cognition in Neurological Disorders. Nutrients 2021, 13 (6), 2099 10.3390/nu13062099. 34205336 PMC8234057

[ref100] Hamamah S.; Aghazarian A.; Nazaryan A.; Hajnal A.; Covasa M. Role of Microbiota-Gut-Brain Axis in Regulating Dopaminergic Signaling. Biomedicines 2022, 10 (2), 436 10.3390/biomedicines10020436. 35203645 PMC8962300

[ref101] Markowiak-Kopeć P.; Śliżewska K. The Effect of Probiotics on the Production of Short-Chain Fatty Acids by Human Intestinal Microbiome. Nutrients 2020, 12 (4), 1107 10.3390/nu12041107. 32316181 PMC7230973

[ref102] Pi Y.; Fang M.; Li Y.; Cai L.; Han R.; Sun W.; Jiang X.; Chen L.; Du J.; Zhu Z.; et al. Interactions between Gut Microbiota and Natural Bioactive Polysaccharides in Metabolic Diseases: Review. Nutrients 2024, 16 (17), 2838 10.3390/nu16172838. 39275156 PMC11397228

[ref103] Han K.; et al. Propionate functions as a feeding state-dependent regulatory metabolite to counter proinflammatory signaling linked to nutrient load and obesity. J. Leukoc. Biol. 2024, 115 (4), 738–749. 10.1093/jleuko/qiae006. 38207130 PMC10980352

[ref104] Belkaid Y.; Hand T. W. Role of the microbiota in immunity and inflammation. Cell 2014, 157 (1), 121–141. 10.1016/j.cell.2014.03.011. 24679531 PMC4056765

[ref105] Li Y.; et al. Sodium butyrate alleviates lead-induced neuroinflammation and improves cognitive and memory impairment through the ACSS2/H3K9ac/BDNF pathway. Environ. Int. 2024, 184, 108479 10.1016/j.envint.2024.108479. 38340407

[ref106] Cheng J.; et al. Gut microbiota-derived short-chain fatty acids and depression: deep insight into biological mechanisms and potential applications. Gen. Psychiatr. 2024, 37 (1), e101374 10.1136/gpsych-2023-101374. 38390241 PMC10882305

[ref107] Carloni S.; Rescigno M. The gut-brain vascular axis in neuroinflammation. Semin. Immunol. 2023, 69, 101802 10.1016/j.smim.2023.101802. 37422929

[ref108] Tang W.; et al. The Impact of Gut Microbiota Disorders on the Blood-Brain Barrier. Infect. Drug Resist. 2020, 13, 3351–3363. 10.2147/IDR.S254403. 33061482 PMC7532923

[ref109] Henry N.; Frank J.; McLouth C.; Trout A. L.; Morris A.; Chen J.; Stowe A. M.; Fraser J. F.; Pennypacker K. Short Chain Fatty Acids Taken at Time of Thrombectomy in Acute Ischemic Stroke Patients Are Independent of Stroke Severity But Associated With Inflammatory Markers and Worse Symptoms at Discharge. Front. Immunol. 2022, 12, 797302 10.3389/fimmu.2021.797302. 35126360 PMC8807638

[ref110] Jaworska J.; et al. Effect of the HDAC Inhibitor, Sodium Butyrate, on Neurogenesis in a Rat Model of Neonatal Hypoxia-Ischemia: Potential Mechanism of Action. Mol. Neurobiol. 2019, 56 (9), 6341–6370. 10.1007/s12035-019-1518-1. 30767185 PMC6682584

[ref111] Mitra S.; et al. Brain modulation by the gut microbiota: From disease to therapy. J. Adv. Res. 2023, 53, 153–173. 10.1016/j.jare.2022.12.001. 36496175 PMC10658262

[ref112] Kang J. W.; et al. Gut microbial metabolism in Alzheimer’s disease and related dementias. Neurotherapeutics 2024, 21 (6), e00470 10.1016/j.neurot.2024.e00470. 39462700 PMC11585892

[ref113] Jenkinson M.; et al. Improved Optimization for the Robust and Accurate Linear Registration and Motion Correction of Brain Images. Neuroimage 2002, 17, 825–841. 10.1006/nimg.2002.1132. 12377157

[ref114] Rohde G.; et al. Estimating intensity variance due to noise in registered images—applications to DTI MRI. Neuroimage 2005, 26, 673–684. 10.1016/j.neuroimage.2005.02.023. 15955477

[ref115] Huang H.; et al. Correction of B0 susceptibility induced distortion in diffusion-weighted images using large-deformation diffeomorphic metric mapping. Magn. Reson. Imaging 2008, 26 (9), 1294–1302. 10.1016/j.mri.2008.03.005. 18499384 PMC2612638

[ref116] Malekian V.; et al. Mitigating susceptibility-induced distortions in high-resolution 3DEPI fMRI at 7T. Neuroimage 2023, 279, 120294 10.1016/j.neuroimage.2023.120294. 37517572 PMC10951962

[ref117] Henriques R. N.; et al. Diffusional Kurtosis Imaging in the Diffusion Imaging in Python Project. Front. Hum. Neurosci. 2021, 15, 675433 10.3389/fnhum.2021.675433. 34349631 PMC8327208

[ref118] Wu M.; et al. Optimum template selection for atlas-based segmentation. Neuroimage 2007, 34, 1612–1618. 10.1016/j.neuroimage.2006.07.050. 17188896

[ref119] Ballanger B.; Tremblay L.; Sgambato-Faure V.; Beaudoin-Gobert M.; Lavenne F.; Le Bars D.; Costes N. A multi-atlas based method for automated anatomical Macaca fascicularis brain MRI segmentation and PET kinetic extraction. Neuroimage 2013, 77, 26–43. 10.1016/j.neuroimage.2013.03.029. 23537938

[ref120] Feng J.; et al. Detection of Alzheimer’s disease using features of brain region-of-interest-based individual network constructed with the sMRI image. Comput. Med. Imaging Graph. 2022, 98, 102057 10.1016/j.compmedimag.2022.102057. 35561640

[ref121] Kim R. E. Y.; et al. Increased Likelihood of Dementia with Coexisting Atrophy of Multiple Regions of Interest. J. Alzheimers Dis. 2024, 97 (1), 259–271. 10.3233/JAD-230602. 38143346

[ref122] Cacioppo J. T.; Tassinary L. G.; Berntson G. Handbook of Psychophysiology; Cambridge University Press, 2007.

[ref123] Shavelson R. J. Statistical Reasoning for the Behavioral Sciences; Citeseer, 1988.

[ref124] Faul F.; et al. G*Power 3: A flexible statistical power analysis program for the social, behavioral, and biomedical sciences. Behav. Res. Methods 2007, 39 (2), 175–191. 10.3758/BF03193146. 17695343

