# Supplementary material for: Interplay of Neuroinflammation and Gut Microbiota Dysbiosis in Alzheimer’s Disease Using Diffusion Kurtosis Imaging Biomarker in 3 × Tg-AD Mouse Models
Source: ACS Chem Neurosci. 2025 Apr 8;16(8):1511–28. doi: 10.1021/acschemneuro.5c00063 (PMC12006996; doi:10.1021/acschemneuro.5c00063)
Supplement: Supplementary file 1 — cn5c00063_si_001.pdf [file cn5c00063_si_001.pdf]

Supplementary Materials

Interplay of Neuroinflammation and Gut Microbiota Dysbiosis in Alzheimer's Disease Using Diffusion Kurtosis Imaging Biomarker in 3×Tg-AD Mouse Models

Lalitha Palanivelu[1], Ching-Wen Chang[2], Ssu-Ju Li[2], Yao-Wen Liang[2], Yu-Chun Lo[3*†], and You-Yin Chen[2,3*†]

[1]International Ph.D. Program in Medicine, College of Medicine, Taipei Medical University, 7F., No. 250, Wuxing St., Xinyi Dist., Taipei 11031, Taiwan
[2]Department of Biomedical Engineering, National Yang Ming Chiao Tung University, No.155, Sec.2, Linong St., Taipei 112304, Taiwan
[3]Ph.D. Program in Medical Neuroscience, College of Medical Science and Technology, Taipei Medical University. 12F., Education and Research Building, Shuang-Ho Campus, No. 301, Yuantong Rd., New Taipei City 23564, Taiwan

[†]These authors have contributed equally to this work

* Correspondence should be addressed to the following:
Yu-Chun Lo, Ph.D.
Ph.D. Program in Medical Neuroscience, College of Medical Science and Technology, Taipei Medical University. 12F., Education and Research Building, Shuang-Ho Campus, No. 301, Yuantong Rd., New Taipei City 23564, Taiwan.
E-mail: aricalo@tmu.edu.tw

You-Yin Chen, Ph.D.
Department of Biomedical Engineering, National Yang Ming Chiao Tung University, No.155, Sec.2, Linong St., Taipei 112304, Taiwan
E-mail: irradiance@so-net.net.tw/youyin.chen@nycu.edu.tw

Note 1: *Tract-based Analysis of White Matter Connectivity Between ROIs in AD Model at Different Time Points*

The **Figure S1** presents a detailed analysis of white matter connectivity changes in an AD model using DTI fiber tract analysis. The data illustrate alterations in major white matter pathways between WT and AD groups at 6-MO and 12-MO. The visualization highlights key tracts connecting the mPFC, HIPP, EC, fornix, STR, and NAc, providing insight into the structural organization of these circuits.

FA measurements are used to assess white matter integrity, with the color-coded fiber reconstructions depicting connectivity differences between groups. Warmer colors in the FA maps indicate higher values, while lower FA in the AD model suggests compromised structural integrity of white matter pathways. The quantified FA data further reveal significant reductions in the AD group at both time points, reflecting progressive degeneration. These findings provide evidence of persistent white matter disruptions in the AD model, potentially contributing to impaired neural communication and cognitive decline associated with the disease.

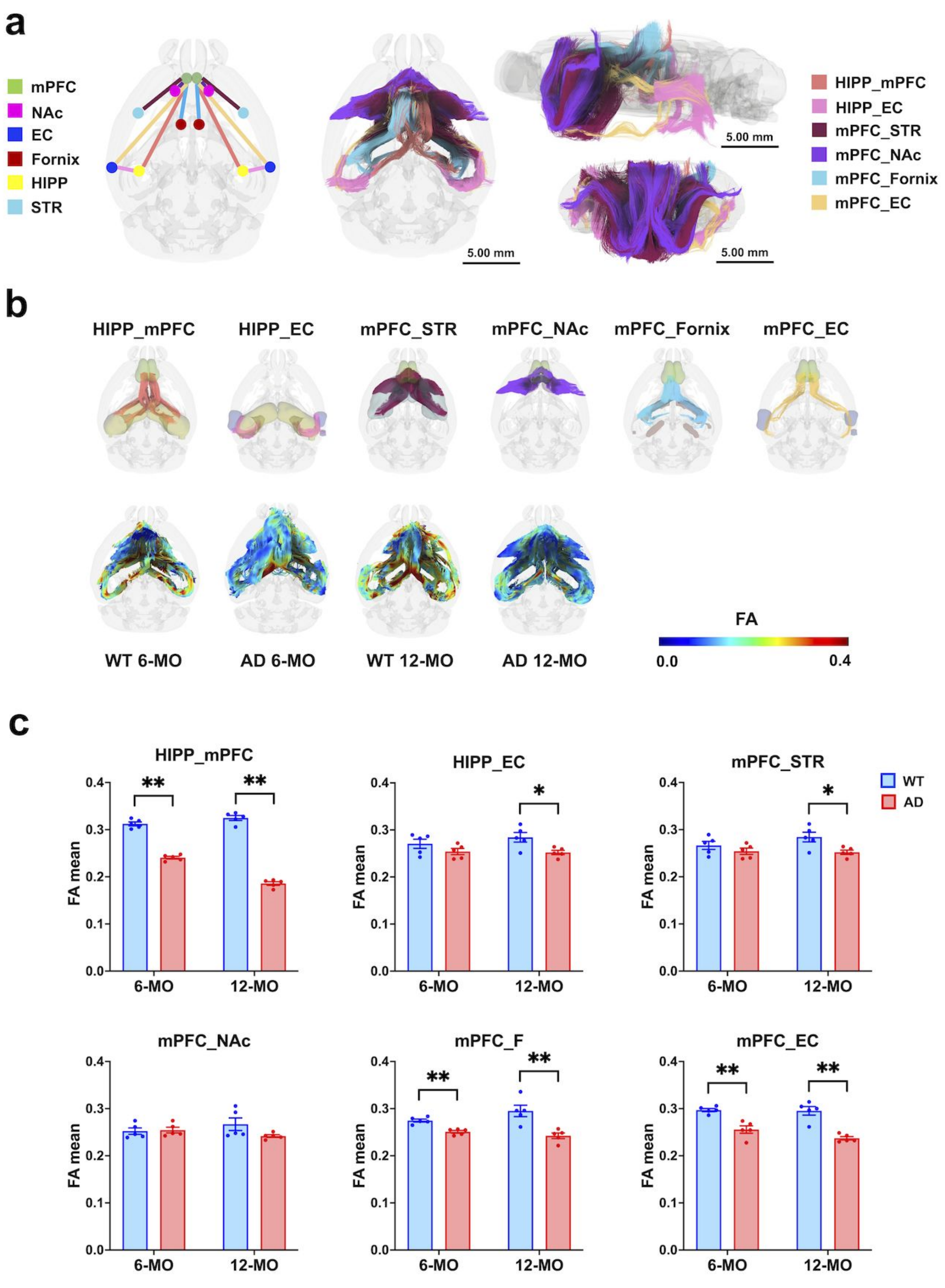

Figure S1. DTI Fiber Tract Analysis of White Matter Connectivity Between ROIs in AD Model at Different Time Points. DTI fiber tract analysis and FA measurements in key white matter pathways between WT and AD groups at 6-MO and 12-MO. (a) illustrates the anatomical distribution of major white matter tracts, with different colors representing connections involving the mPFC, NAc, EC, fornix, HIPP and STR. The leftmost image provides a schematic overview of these pathways, while the other images offer 3D reconstructions of fiber projections. (b) compares white matter tracts between WT and AD groups at both time points. The top row displays tract reconstructions for HIPP_mPFC, HIPP_EC, mPFC_STR, mPFC_NAc,

mPFC_Fornix, and mPFC_EC, while the bottom row highlights FA differences using a color gradient, with warmer colors indicating higher FA values. (c) quantifies FA in these tracts, showing significant reductions in the AD group compared to WT at both 6-MO and 12-MO. Statistical analysis (Mann–Whitney U test, FDR-adjusted): $^{*}p < 0.05$, $^{**}p < 0.01$. Data are shown as mean ± SEM, with individual points representing sample sizes.

Note 2: *LC-MS/MS analysis of SCFAs from animal serum samples*

Table S1 provides quantitative information on derivatized SCFAs obtained from animal serum samples analyzed through LC-MS/MS. **Table S1** summarizes the key parameters used in the analysis, including precursor/product ion mass-to-charge ratios (*m/z*), isotope-labelled internal standards, retention times, collision energies, and tube lens voltages for each analyte.

Table S1. Quantitative information on derivatized SCFAs

Analyte	*Precursor/product ions (m/z)*	*Isotope-labelled precursor/product ions (m/z)*	*Retention time (min)*	*Collision energy (V)*	*Tube lens (V)*
Formic acid	180/137	186/143	4.70	20	29
Acetic acid	194/137	200/143	5.22	23	39
Propionic acid	208/137	214/143	7.18	29	41
Butyric acid	222/137	228/143	9.12	21	40
Isobutyric acid	222/137	228/143	8.87	21	40
2-methyl butyric acid	236/137	242/143	10.58	23	50
Valeric acid	236/137	242/143	11.22	23	50
Isovaleric acid	236/137	242/143	10.86	23	50
3-methylvaleric acid	250/137	256/143	12.62	33	50

The analytes include formic acid, acetic acid, propionic acid, butyric acid, isobutyric acid, 2-methyl butyric acid, valeric acid, isovaleric acid, and 3-methylvaleric acid. Isotope-labelled SCFAs were used as internal standards to enhance the accuracy and reliability of quantification. Retention times range from 4.70 to 12.62 min, and the optimized collision energy and tube lens voltage settings ensure effective ion fragmentation and signal detection for precise measurements.

Note 3: *Effect Sizes and Statistical Power Across Behavioral, Neuroanatomical, Immunohistochemical, Cytokine, and SCFA Assessments Between two Groups at Different Time Points*

This collection of tables presents quantitative analyses of effect sizes and statistical power across multiple experimental domains, including behavioral assessments, neuroimaging (DKI and DTI), immunohistochemistry, inflammatory cytokine levels, gut microbiota composition, and SCFA concentrations in a comparative study between two groups at different developmental time points (6-MO and 12-MO). Effect sizes provide a measure of the magnitude of differences observed between groups, while statistical power indicates the likelihood of detecting a true effect.

Table S2 presents effect sizes and statistical power for the T-maze test at 6-MO and 12-MO of age. **Table S2** summarizes the test assessing spatial learning and working memory, with results showing strong effect sizes (1.299 at 6-MO and 2.253 at 12-MO) and high statistical power (> 0.95), suggesting robust group differences in cognitive performance.

Table S2. Effect Sizes and Statistical Power for T-maze Test Between Groups at Different Time Points

Experiment	*Time point*	*Effect size*	*Power*
T-Maze Test	6-MO	1.299	0.955
	12-MO	2.253	0.975

Table S3 summarizes effect sizes and statistical power for DKI indices across various brain regions at 6-MO and 12-MO. **Table S3** summarizes the parameters analyzed include AK, MK, and RK in regions such as the mPFC, EC, HIPP, fornix, NAc, and STR. RK showed the highest effect sizes across time points, particularly in the mPFC (1.087 at 6-MO and 0.825 at 12-MO), indicating potential microstructural alterations.

Table S3. Effect Sizes and Statistical Power for DKI analysis Between Groups at Different Time Points

Experiment	*Time point*	*DKI index*	*Brain region*	*Effect size*	*Power*
DKI Indexes	6-MO	AK	mPFC	0.182	0.950
			EC	0.144	0.950
			Fornix	0.169	0.950
			HIPP	0.119	0.950
			NAc	0.184	0.950
			STR	0.111	0.950
		MK	mPFC	0.376	0.950
			EC	0.363	0.950
			Fornix	0.364	0.950
			HIPP	0.409	0.950
			NAc	0.359	0.950
			STR	0.250	0.950
		RK	mPFC	1.087	0.958
			EC	0.751	0.950
			Fornix	0.771	0.954
			HIPP	0.938	0.954
			NAc	0.425	0.951
			STR	0.455	0.950
	12-MO	AK	mPFC	0.239	0.950
			EC	0.200	0.950
			Fornix	0.161	0.950
			HIPP	0.118	0.950
			NAc	0.303	0.950
			STR	0.158	0.950
		MK	mPFC	0.367	0.950
			EC	0.660	0.953
			Fornix	0.315	0.950

			HIPP	0.634	0.951
			NAc	0.440	0.951
			STR	0.416	0.950
		RK	mPFC	0.825	0.952
			EC	0.019	0.950
			Fornix	1.096	0.952
			HIPP	0.585	0.950
			NAc	1.296	0.954
			STR	0.622	0.951

Table S4 presents the effect sizes and statistical power for DTI-based fiber tract analysis, specifically assessing FA across key white matter connections at 6-MO and 12-MO. **Table S4** summarizes the analyzed tracts include HIPP_mPFC, HIPP_EC, mPFC_STR, mPFC_NAc, mPFC_Fornix, and mPFC_EC. Effect sizes were generally low across all tracts, with the highest observed at 12-MO in the HIPP_mPFC connection (0.277). Despite small effect sizes, statistical power remained constant at 0.950 for all comparisons, indicating that the study design was adequately powered to detect even subtle group differences. These results suggest minimal alterations in FA between groups across the assessed time points.

Table S4. Effect Sizes and Statistical Power for DTI Fiber Tract Analysis Between Groups at Different Time Points

Experiment	*Time point*	*DTI index*	*Brain region*	*Effect size*	*Power*
DTI fiber tract analysis	6-MO	FA	HIPP_mPFC	0.143	0.950
			HIPP_EC	0.032	0.950
			mPFC_STR	0.024	0.950
			mPFC_NAc	0.004	0.950
			mPFC_Fornix	0.047	0.950
			mPFC_EC	0.082	0.950
	12-MO	FA	HIPP_mPFC	0.277	0.950
			HIPP_EC	0.064	0.950
			mPFC_STR	0.064	0.950
			mPFC_NAc	0.050	0.950
			mPFC_Fornix	0.105	0.950
			mPFC_EC	0.116	0.950

Table S5 provides effect sizes and statistical power for immunohistochemical markers of astrocytes (GFAP^{+}) and microglia (IBA-1^{+}) across brain regions. Notably, at 12-MO, IBA-1^{+} expression in the mPFC showed an effect size of 9.200, indicating substantial differences in microglial activation. Similarly, GFAP^{+} levels exhibited strong effect sizes across multiple regions, with the highest in the mPFC (6.760) and EC (6.880) at 12-MO.

Table S5. Effect Sizes and Statistical Power for IHC analysis Between Groups at Different Time Points

Experiment	*Time point*	*Brain region*	*Cell type*	*Effect size*	*Power*
IHC Analysis	6-MO	mPFC	GFAP^{+}	2.320	0.964
			IBA-1^{+}	1.680	0.961
		EC	GFAP^{+}	1.720	0.967
			IBA-1^{+}	4.000	0.988
		Fornix	GFAP^{+}	0.560	0.950
			IBA-1^{+}	0.200	0.950
		HIPP	GFAP^{+}	3.560	0.969
			IBA-1^{+}	0.200	0.950
		NAc	GFAP^{+}	2.560	0.985

			IBA-1$^+$	2.640	0.984
		STR	GFAP$^+$	4.040	0.998
			IBA-1$^+$	0.400	0.950
	12-MO	mPFC	GFAP$^+$	6.760	0.988
			IBA-1$^+$	9.200	0.999
		EC	GFAP$^+$	6.880	0.989
			IBA-1$^+$	6.440	0.982
		Fornix	GFAP$^+$	7.240	0.983
			IBA-1$^+$	5.560	0.982
		HIPP	GFAP$^+$	6.280	0.978
			IBA-1$^+$	5.520	0.952
		NAc	GFAP+	1.400	0.953
			IBA-1+	4.900	0.999
		STR	GFAP+	5.640	0.956
			IBA-1+	4.680	0.998

Table S6 summarizes effect sizes and statistical power for inflammatory cytokines (IL-1β, IL-6, IFN-γ, and TNFα). Effect sizes were highest for IL-1β and IL-6 at both time points (>15.000), indicating substantial inflammatory differences between groups. Statistical power for all cytokine measurements was near 1.000, reflecting highly reliable findings.

Table S6. Effect Sizes and Statistical Power for Inflammatory Cytokines Analysis Between Groups at Different Time Points

Experiment	*Time point*	*Cytokine*	*Effect size*	*Power*
Inflammatory Cytokines Analysis	6 MO	IL-1β	15.604	1.000
		IL-6	19.736	1.000
		IFN-γ	2.952	0.977
		TNFα	3.140	0.987
	12 MO	IL-1β	15.396	1.000
		IL-6	14.16	1.000
		IFN-γ	12.34	0.999
		TNFα	17.78	1.000

Table S7 presents effect sizes and statistical power for gut microbiota composition, specifically **Table S7** summarizes the relative abundance of *Firmicutes* and *Bacteroidetes*, as well as the *F/B* ratio. The highest effect size was observed for *Firmicutes* at 6-MO (19.155), indicating strong microbial differences between groups.

Table S7. Effect Sizes and Statistical Power for Gut Microbiota Analysis Between Groups at Different Time Points

Experiment	*Time point*	*SCFA*	*Effect size*	*Power*
Gut Microbiota Analysis	6 MO	*Fermicutes*	19.155	1.000
		Bacteroidetes	10.001	0.950
		F/B	0.276	0.950
	12 MO	*Fermicutes*	11.004	0.999
		Bacteroidetes	15.976	1.000
		F/B	0.523	0.950

Table S8 provides effect sizes and statistical power for SCFA concentrations, including acetate, propionate, isobutyrate, butyrate, isovalerate, and valerate. At age of 12-MO, acetate (8.600) and propionate (8.920) showed the largest effect sizes, highlighting significant group differences in microbial-derived

metabolites. These findings collectively suggest substantial group differences across multiple domains, with strong effect sizes and high statistical power, reinforcing the reliability of the observed trends in behavioral, neuroanatomical, immune, and metabolic assessments.

Table S8. Effect Sizes and Statistical Power for SCFA Analysis Between Groups at Different Time Points

Experiment	*Time point*	*SCFA*	*Effect size*	*Power*
**SCFA Analysis**	6 MO	Acetate	4.460	0.996
		Propionate	8.232	0.998
		Isobutyrate	2.352	0.958
		Butyrate	3.544	0.968
		Isovalerate	2.000	0.969
		Valerate	1.360	0.957
	12 MO	Acetate	8.600	0.998
		Propionate	8.920	0.999
		Isobutyrate	3.340	0.951
		Butyrate	4.292	0.994
		Isovalerate	2.396	0.963
		Valerate	2.124	0.961
